# Volumetric Additive Manufacturing for Cell Printing: Bridging Industry Adaptation and Regulatory Frontiers

**DOI:** 10.1021/acsbiomaterials.4c01837

**Published:** 2025-01-02

**Authors:** Vidhi Mathur, Vinita Dsouza, Varadharajan Srinivasan, Kirthanashri S Vasanthan

**Affiliations:** † Manipal Centre for Biotherapeutics Research, Manipal Academy of Higher Education, Manipal, 576104 Karnataka, India; ‡ Department of Civil Engineering, Manipal Institute of Technology, Manipal Academy of Higher Education, Manipal, 576104 Karnataka, India

**Keywords:** Volumetric additive manufacturing, light-based printing, tomography, additive manufacturing

## Abstract

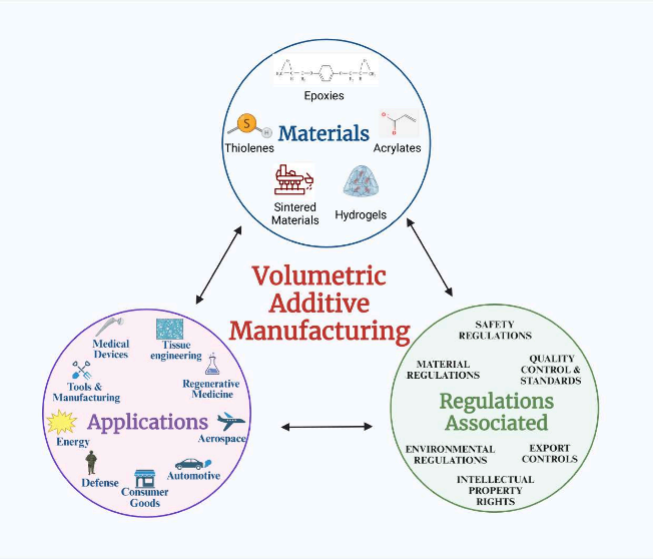

Volumetric additive manufacturing (VAM) is revolutionizing the field of cell printing by enabling the rapid creation of complex three-dimensional cellular structures that mimic natural tissues. This paper explores the advantages and limitations of various VAM techniques, such as holographic lithography, digital light processing, and volumetric projection, while addressing their suitability across diverse industrial applications. Despite the significant potential of VAM, challenges related to regulatory compliance and scalability persist, particularly in the context of bioprinted tissues. In India, the lack of clear regulatory guidelines and intellectual property protections poses additional hurdles for companies seeking to navigate the evolving landscape of bioprinting. This study emphasizes the importance of collaboration among industry stakeholders, regulatory agencies, and academic institutions to establish tailored frameworks that promote innovation while ensuring safety and efficacy. By bridging the gap between technological advancement and regulatory oversight, VAM can unlock new opportunities in regenerative medicine and tissue engineering, transforming patient care and therapeutic outcomes.

## Introduction

In the realm of regenerative medicine, the creation of laboratory-generated 3D living constructs that replicate the functionality of human tissues and organs is a pivotal aspiration. Such constructs hold immense promise for advancing regenerative medicine and facilitating the development of sophisticated *in vitro* models crucial for drug discovery, toxicology testing, and precision medicine.^1,2^ Advancements in technologies paves way to develop scaffolds that have tailored properties and are crucial for regeneration.^3^ Various types of 3D printing technologies are available, including extrusion-based, stereolithography-based, laser, inkjet-based, and vat polymerization-based, while extrusion- based 3D printer is most commonly utilized for bioprinting and being used for articular cartilage repair, bone repairment and healing, etc.^4^ All of these printing technologies use a layer-by-layer approach apart from the VAM, where an object is formed by irradiating an entire volume of photosensitive resin. The major drawbacks of layer-by-layer approach being, the use of a suitable biomaterial, viscosity parameters, the challenge of supporting and retaining the structure and subsequent removal, leading to potential loss of structural integrity under load.^5^ Despite there being significant improvements in different 3D printing techniques, challenges persist regarding printing duration and geometric constraints. In contrast volumetric- based 3D printing offers a promising alternative, which employs a nozzle-free method, to print the object by utilizing volumetric image modalities like computed tomography (CT).^6^ We have mentioned a few differences between traditional 3D printing and VAM in Table 1. CT used in biomedical imaging served as the inspiration for tomographic 3D printing.^7^ In VAM a photo resin is exposed to light from several angles to create a 3D object. Digitally produced light patterns from a digital light processing module (DLPM) are used to ease this irradiation. The light patterns are spatially projected onto a revolving glass vial containing the resin. Through cross-linking processes, the controlled exposure to light causes the resin to solidify locally, because VAM involves rotating movement, high-viscosity resins must be used to guarantee the integrity of the structures that are created.^8^ VAM represents progressive advancement beyond sequential biofabrication methodologies, thereby unlocking novel opportunities for rapid additive manufacturing across various domains such as tissue engineering, regenerative medicine, personalized pharmaceutical assessment, soft robotics, and beyond.^9^ The main advantage of VAM is that it offers a rapid printing speed and can overcome the geometric and surface quality, and the viscosity of the biomaterials, as VAM depends on the resin with low viscosity limitations encountered by the layer-by-layer approach in the vat polymerization techniques.^10^ Similarly, the upcoming state of the art technology is 4D printing which has the ability to manipulate the material properties and geometries and enhances the performance of the structures.^11^

**Table 1:** Differences between Traditional 3D printing and Volumetric Additive Manufacturing^137,138^

Traditional 3D printing	Volumetric Additive Manufacturing
Creates objects that are not possible by subtractive methods in a layer-by-layer fashion	This applies energy to selective points of the material to generate a 3D object, it is a layer less method
Multiple layered object printing is very time-consuming	The printing proceeds quickly as the entire object is simultaneously solidified
Prints with a nozzle	Nozzle not required
Needs support structures while printing overhanging models	Support structures are not required
Complex structures with intricate geometries are difficult to print	Structures with complicated intricate geometries are printed easily
Materials such as ceramics, metals and common plastics have been used	Specialized materials such as photosensitive resins, polymers, ceramics, and metal powders are being used
There is an increased material wastage, due to the support structures and during the layering process	The material wastage is lower due to the accurate solidification of the volume
Resolution depends on the nozzle size and layer thickness and it may vary	Resolution is high, it also depends on the technology employed
Equipment cost can vary	Requires specialized equipment of high cost
It is suitable for variety of applications such as aerospace and industrial manufacturing	Most frequently used in many industries, but its layering constraints may cause restrictions
Advancing continuously in materials and techniques of printing and its established well	Research is still in early stages of development and ongoing

**Figure 1:**
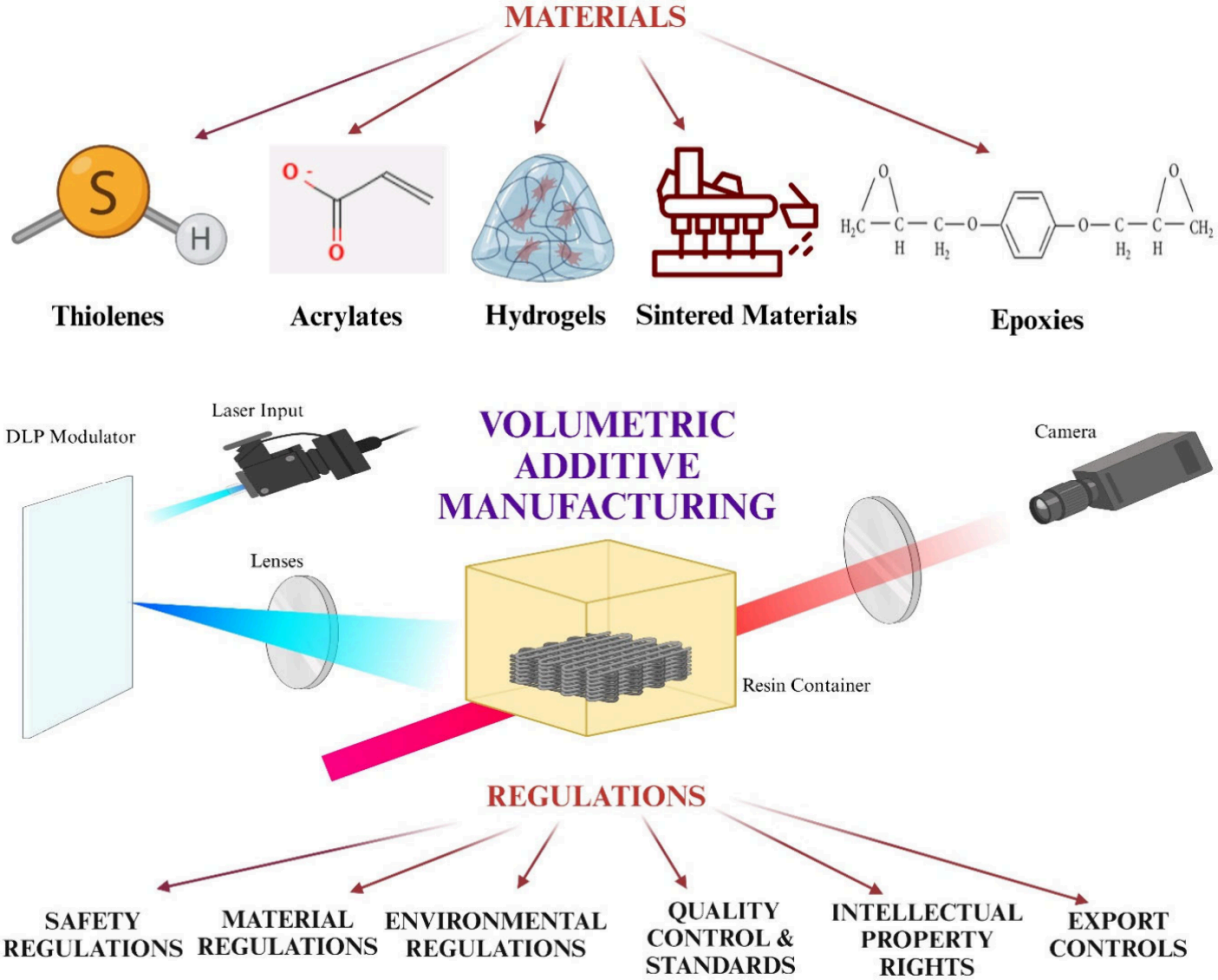
Overview of VAM: Materials and regulations.

This review provides an overview of VAM, detailing its core principles and methodologies. Subsequently, the evaluation of the many materials employed within VAM, is followed by an exploration of the myriad applications. Furthermore, the review offers insights into the future outlook of VAM, including a concise discussion on its limitations, potential advancements, and ongoing challenges. The review distinguishes itself by providing a holistic perspective on VAM and not only emphasizing the fundamentals principle, materials, and applications but also addressing critical aspects of environmental and occupational safety. This work bridges the gap between technology implementation and responsible implementation making it relevant for advancing discussions on sustainability, regulatory compliance and implications of VAM. The overview of VAM process with its materials and regulations is shown in Figure 1.

**Figure 2:**
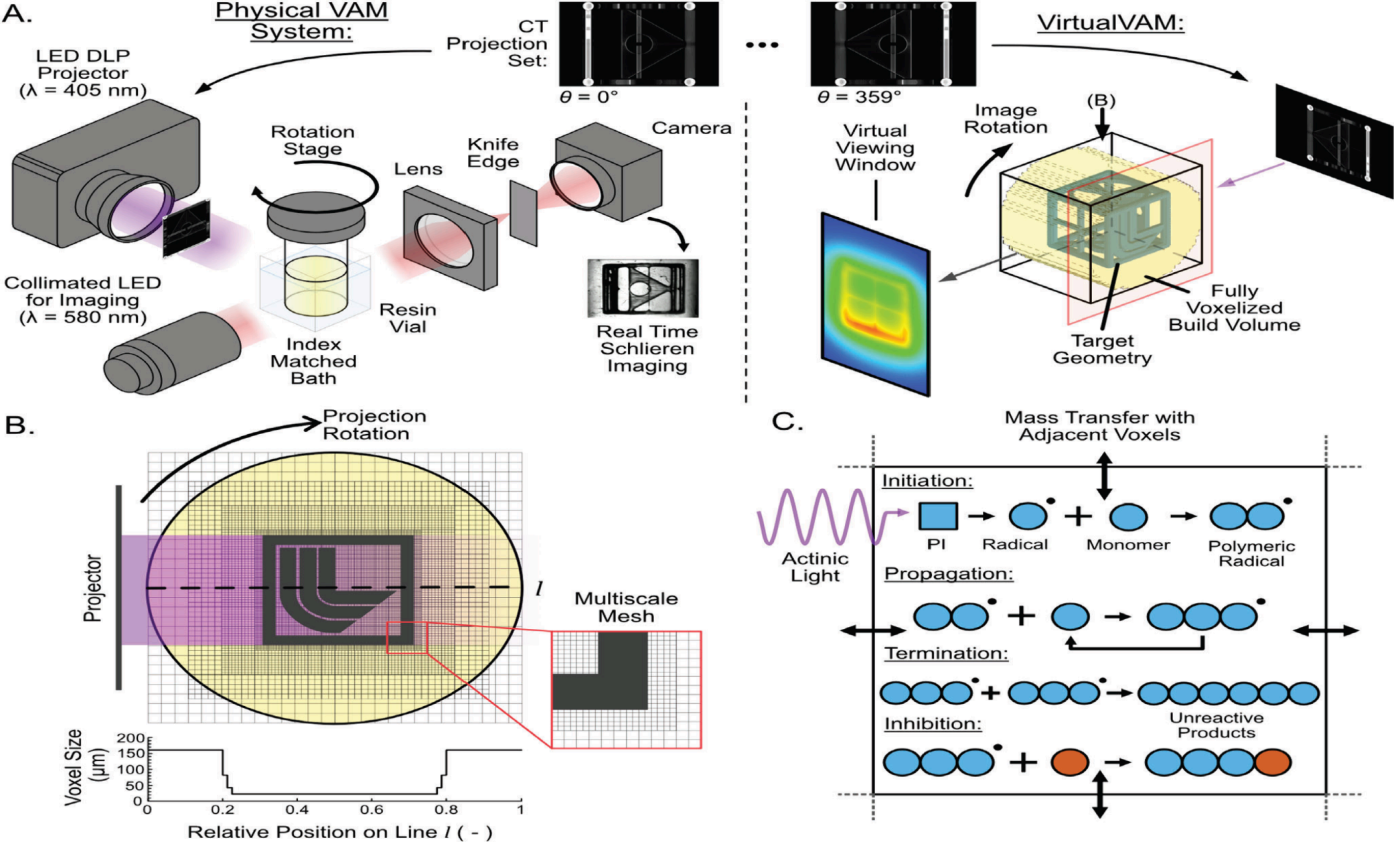
VirtualVAM simulates Reaction and Diffusion in all Voxels in VAM A) Schematic of tomographic VAM with orthogonal Schlieren imaging setup used in this work, along with a schematic of the VirtualVAM simulation framework with associated CT projections, illustrative geometry, and data extraction, B) Slice of the multiscale voxelization used in VirtualVAM simulations and plot of voxel size in the domain along the vial diameter, C) Photopolymerization reactions and mass transfer modeled in each voxel in the simulations, the equations for these effects are given in the Polymerization Reaction and Transport Section. Rights and permission from^23^ under the Creative Commons CC-BY-NC-ND license,.

## Adaptive Light Superposition

The initial volumetric additive manufacturing (VAM) system, introduced by Shusteff et al. in 2017, marked a significant advancement in the field by allowing the rapid production of intricate, nonrepeating 3D structures within a time frame of 1–10 s.^9^ This was accomplished using a resin with nonlinear polymerization initiation properties and by combining three laser beams, each with a wavelength of 532 nm. This approach utilized the threshold behavior of photopolymer resins, preventing polymer formation from a single beam exposure and permitting polymerization only when a voxel experienced the simultaneous intersection of all three beams. Within the scope of this review, the exposure dose distribution function (EDDF) was generated via this concurrent exposure, capitalizing on the threshold behavior of the resin.

**Figure 3:**
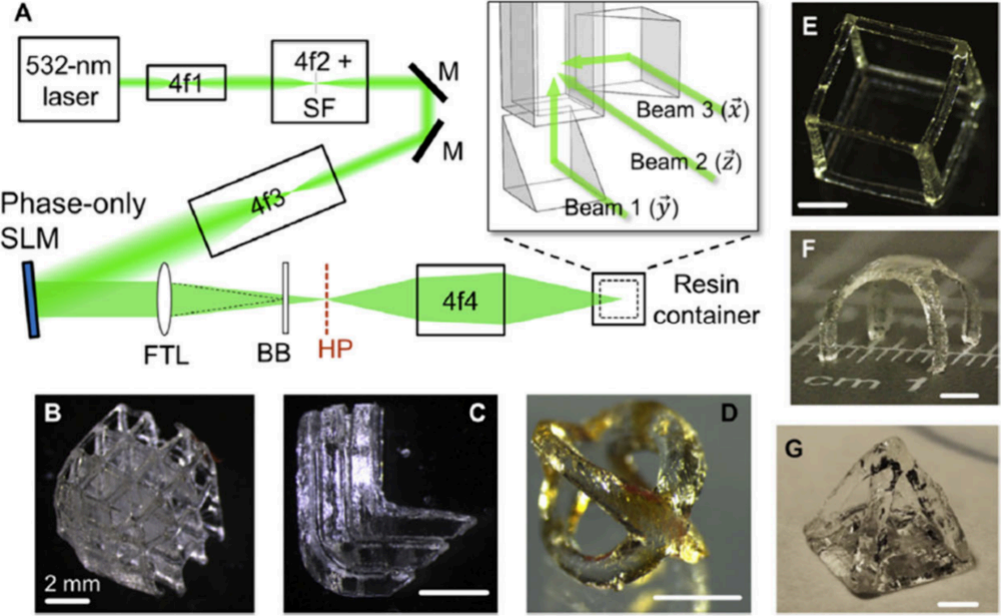
a) Additive beam superposition optics configuration and system schematic. b–g) Examples of printed parts using this light dosage method. Scale bars 2 mm [Shusteff et al., ref (9)]. (Figure reused under Creative commons license).

**Figure 4:**
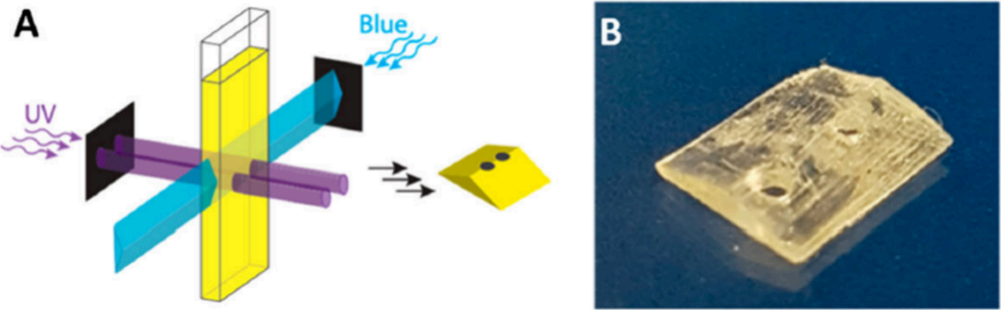
a) Subtractive light superposition optics configuration and system schematic, demonstrating perpendicular blue and near-UV irradiation of the developed resin. b) Example of the printed part using this light dosage method [Vander Laan et al. 2019, REF^10^].

The volumetric exposure method (VEM) involved generating a digital hologram through Fourier transform holography, which was subsequently divided into multiple 2D patterns. Figures 3b–g depict several printed components with complex geometries, while Figure 4a demonstrates the optical setup, showcasing the intersecting beams,

To successfully operate the VAM system, it was crucial to synchronize various properties of the optics and resin formulation. The axial resolution in a single-beam optical setup is notably lower compared to the lateral resolution, necessitating adjustments in each beam’s lateral intensity to offset the limited axial resolution of the other beams. Additionally, the authors examined the influence of molecular oxygen (O_2_) on the free radical polymerization process, as O_2_ is commonly present in resins and often requires purging. Importantly, they discovered that the presence of molecular O_2_ in the resin could be utilized to achieve a nonlinear initiation process (Figure 4).

### Subtractive light Superposition

In 2019, Van der Laan et al. introduced a novel approach utilizing subtractive light superposition, termed dual-wavelength volumetric polymerization.^10^ This technique made use of specific light wavelengths to both initiate and inhibit polymerization within the resin vat. The successful application of subtractive light superposition was achieved through a combination of photoinitiators, camphorquinone (CQ), and Ethyl 4-(dimethylamino)benzoate (EDAB), along with the photoinhibitor butyl nitrite in the resin formulation. The photoinitiators triggered polymerization using visible light, while the photo inhibitor, activated by near-UV wavelengths, limited the growth of polymer chains. Butyl nitrite was chosen as a photo inhibitor due to its moderate absorbance in the near-UV range and weak absorbance in the blue light spectrum, which complemented the absorption characteristics of CQ.

In the context of this Perspective, the VEM step involves slicing the digital model into individual cross sections. The exposure dose distribution function (EDDF) is produced using two projectors, each emitting a different wavelength, and digital micromirror devices (DMDs) to pattern the light into these cross sections, as illustrated in Figure 4A. This process results in the final printed object, accomplished through a combination of inhibition and initiation reactions, as shown in Figure 4B.

Compared to other VAM techniques, the exploration of subtractive light superposition is relatively limited in the literature, making it the least frequently utilized method among those discussed in this review. As a result, only a narrow range of geometries and precision capabilities have been demonstrated, particularly in comparison to the broader applications observed in other VAM approaches. This discrepancy underscores the need for further research into subtractive light superposition to better understand its potential and constraints. Nonetheless, this innovative approach to 3D printing offers new opportunities for research and development in VAM, illustrating how EDDF can induce both inhibition and initiation reactions to create the final printed structure.

## Tomographic Volumetric Additive Manufacturing

**Figure 5:**
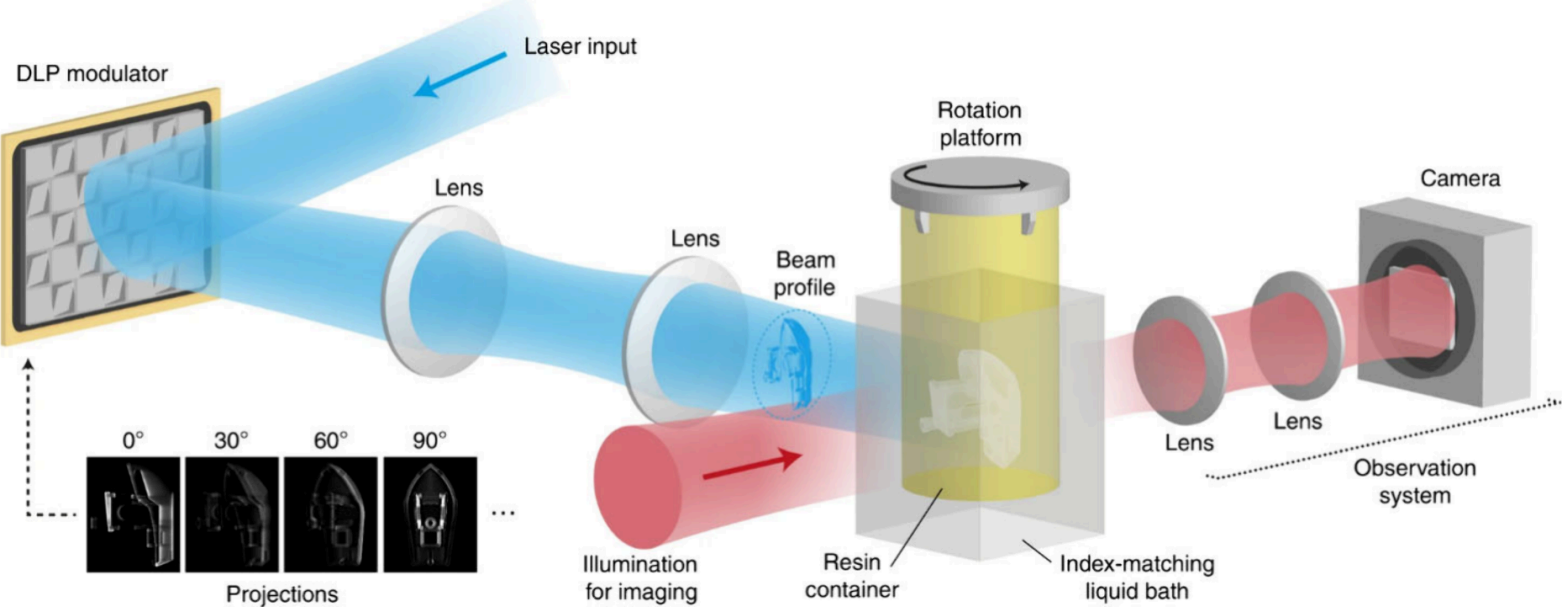
Experimental setup for high-resolution tomographic printing (Loterie et al. 2020, ref (7)) (Figure reused under Creative commons license).

**Figure 6:**
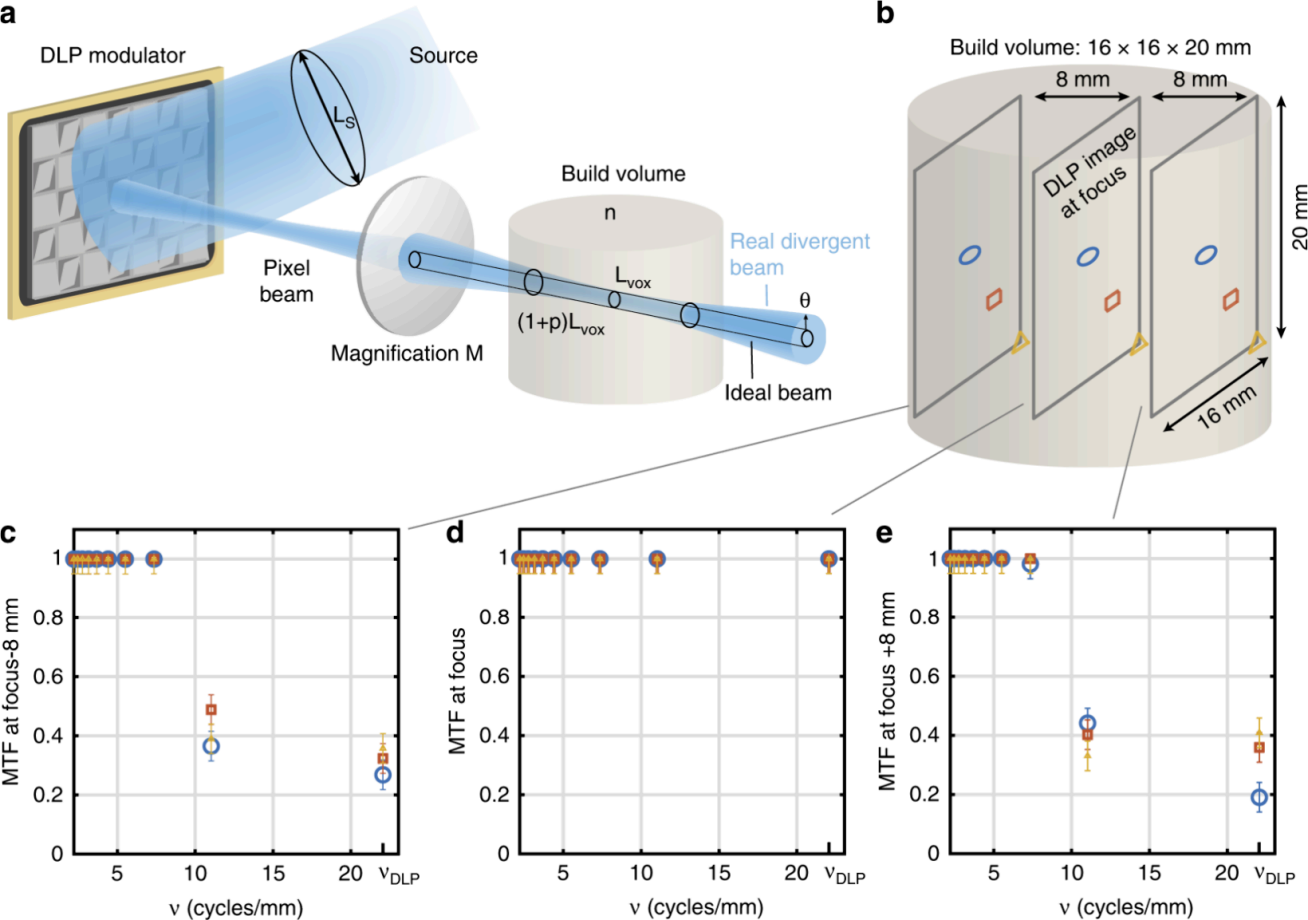
a) étendue-limited optical resolution. b) Experimental measurement points on-axis (blue circle), at midfield, (red square) and edge of field (yellow triangle) of the modulation transfer function (MTF) in the build volume of our tomographic printer. c) Experimental MTF as a function of the spatial frequency 8 mm ahead of the focal plane, d) at focus and e) 8 mm after focus. The error bars represent the standard deviation of five repeated measurements at a point at the edge-of-field point at focus, and the error value for the other points were assumed to be the same (see Supplementary Note 3) (Loterie et al. 2020, ref no 7) (Figure reused under Creative commons license)

**Figure 7:**
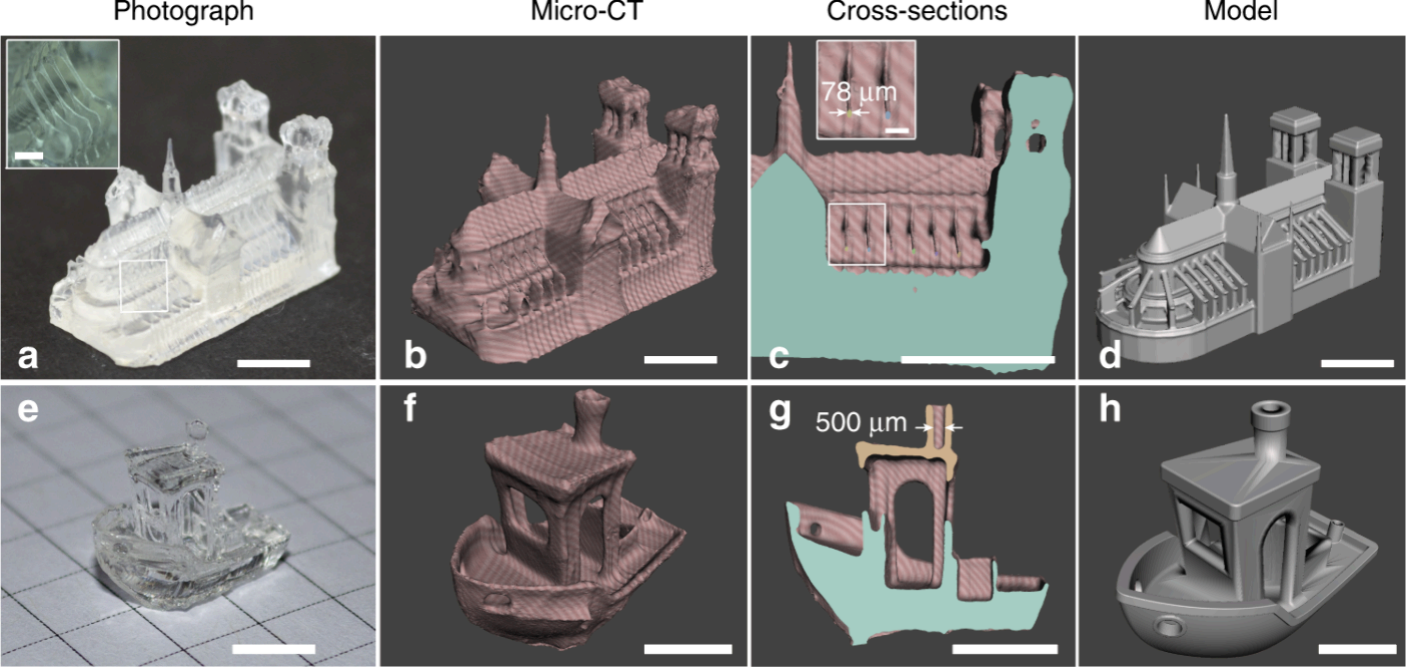
a) Photograph, b) micro-CT rendering, c) micro-CT cross-section, and d) original model for Notre-Dame. A video recording of the printing of Notre Dame is available as Supplementary Movie 3. e) Photograph, f) micro-CT rendering, g) micro-CT cross-section, and h) original model for 3DBenchy. Scale bars: 5 mm. In the inset of a) the scale bar is 1 mm. In the inset of c) the scale bar is 0.5 mm (Loterie et al. 2020, ref no 7) (Figure reused under Creative commons license).

**Figure 8:**
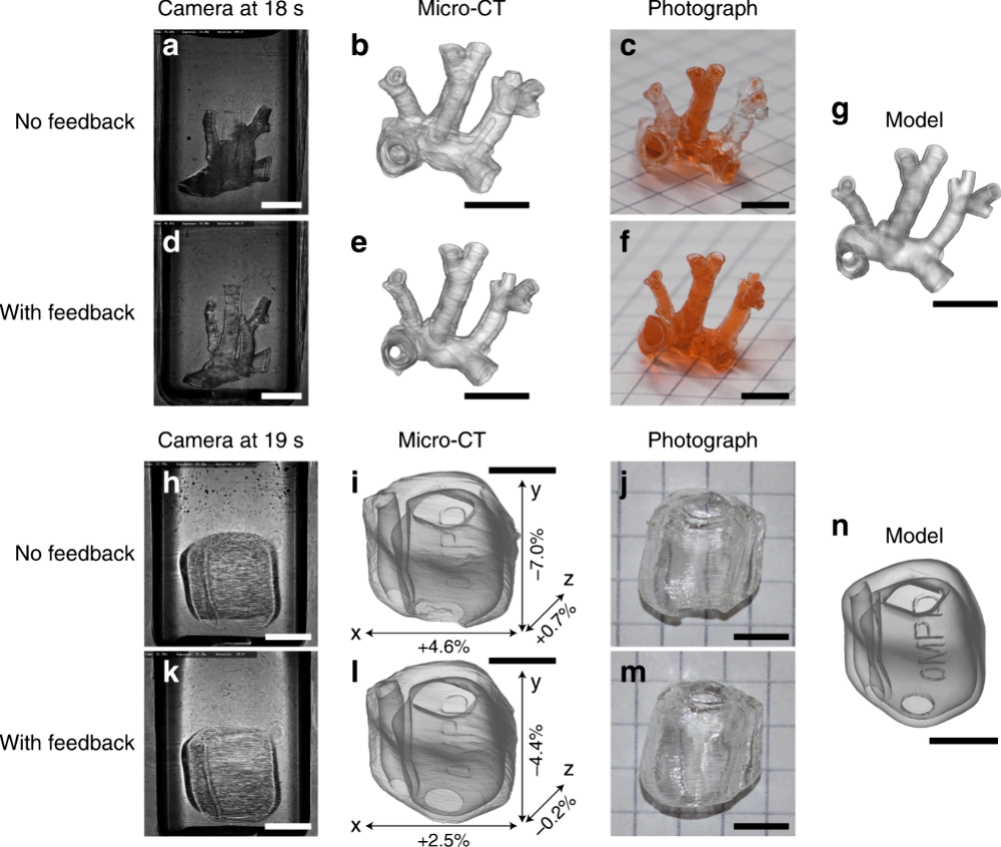
a Video snapshot during the printing of the artery without feedback. b Micro-CT scan of the printed artery (without feedback), rendered with transparency to show the occlusions. c Photograph of the printed artery (without feedback) perfused with a red dye, to visualize the open channels. d–f Corresponding data for the artery printed with feedback. g Digital model of the printed artery. A comparative video recording of the artery prints is available as Supplementary Movie 4. h Video snapshot, (i) micro-CT rendering, and j photograph of the hearing aid model without feedback. k–m Corresponding data for the hearing aid with feedback. n Hearing aid model. i and l show the deviation of the printed parts with respect to the digital model n, as measured by the micro-CT. The scale bars are 5 mm (Loterie et al. 2020, ref no 7) (Figure reused under Creative commons license)

**Figure 9:**
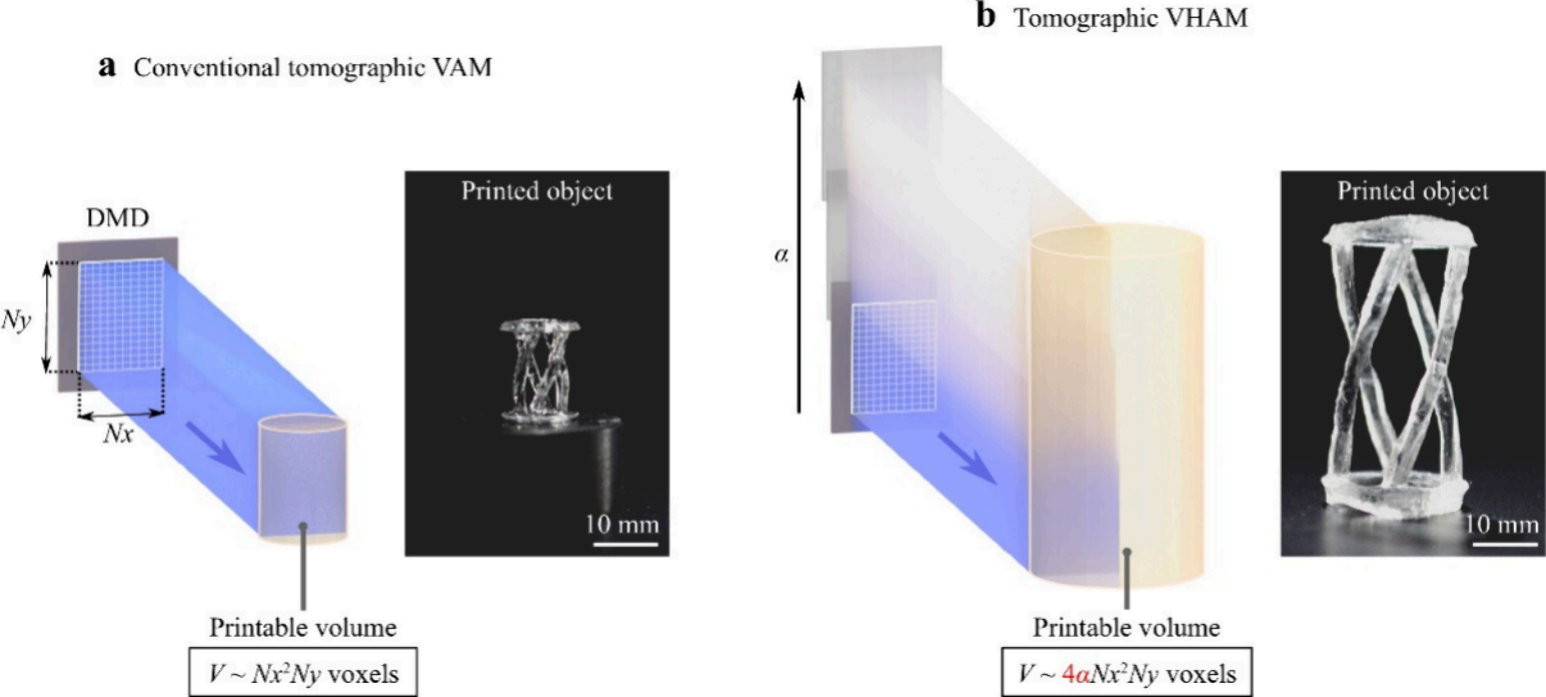
Different tomographic VAM. a Conventional tomographic volumetric additive manufacturing. b Tomographic volumetric helical additive manufacturing. Rights and permission from^27^https://creativecommons.org/licenses/by/4.0/.

The tomographic volumetric AM utilizes the irradiation of an entire photosensitive resin by tuning the absorption length of the resin (Figure 5 & Table 2). Solidification of the whole 3D object occurs simultaneously where the thin slices of resin are cured sequentially.^7^ The liquid photopolymer is irradiated from multiple angles forming dynamic light patterns.^12,13^ The concept of tomographic VAM is based on computerized axial tomography, which is utilized for imaging in medicine.^14^ CT provides images of transverse sections of the tissues and organs and helps in reconstruction of the projections taken at various angles. The tomographic VAM is inspired by CT but in reverse, where the resin is filled in cylinder and rotated continuously and light patterns are produced from digital light processing projector. The patterns are registered and viewed at different angles and then computed using Radon transform which is same as used in CT scan.^15^ Post reaching the gelation threshold, polymerization reaction takes place, which solidifies the liquid resin into solid polymer by chain reaction. The computation of the light dose to be projected is important and is done using filtered back projection algorithm.^16^ The projections at different angles are computed, and the intensity of each pixel is calculated according to Radon transform, which is basically computing along the same angle as viewpoint projection. The tomographic volumetric printers are built to have telecentric chief rays and a minimum spread of light. These requirements are fulfilled either by having digital micromirror device with ultraviolet optical source or another commonly used combination is UV LED for illumination^17^ as shown in Figure 6 ,Figure 7, Figure 8, and Figure 9.

**Table 2:** Tomographic Additive Manufacturing Method

Category	Description
Principle	TVAM is based on computed tomography (CT) scanning techniques. A light source projects 2D patterns from multiple angles, building a 3D object in a single exposure.
Process	Multiple 2D cross-sectional images are projected into a photosensitive material. These images intersect at different angles to form a 3D object within the resin.
Light Source	Typically a laser, UV light, or visible light is used to polymerize the resin. The light patterns are projected simultaneously or sequentially.
Material	Photopolymer resins that can be polymerized by light exposure (such as acrylates or epoxies) are commonly used.
Resolution	Achieves fine resolution, typically in the range of microns, depending on the precision of the light projection and the optics used.
Build Speed	Significantly faster than traditional layer-by-layer methods, as the entire object is constructed simultaneously, rather than one layer at a time.
Key Advantage	Eliminates the need for support structures, making it suitable for highly complex and overhanging geometries.
Challenges	High complexity in the projection system. Limited to certain photosensitive materials. Difficulty in achieving homogeneous light penetration.
Applications	Biomedical implants and scaffolds, Microfluidic devices, High-precision prototyping in aerospace and electronics.
Commercial Availability	Still in the early research and development stage, but emerging rapidly. Some startups and research laboratories are focusing on commercializing the technology.
Advantages over Traditional AM	Faster build times, Ability to create more complex internal geometries, No need for postprocessing to remove supports.
Current Research Trends	Improving material selection, Enhancing light-based control for higher precision, Addressing scalability issues for larger objects.
Limitations	Limited to photopolymerizable materials, Expensive light projection systems, Less mature than traditional AM processes in terms of industrial adoption.
Future Potential	Broader adoption in medical and aerospace sectors, Integration with hybrid manufacturing systems, Use in personalized medicine and custom devices.

Optomechanical setup consists of light source (laser or LED, which are dependent on the photo initiator used), modulator, projection system and rotational stage.^18−20^ It is important that the light source selected should have less divergence and have good resolution. Modulator consists of a digital micromirror that helps in calculating the pattern when the vial is rotated. The vials used for tomographic printing are properly sealed to avoid any contamination.

Webber et al. worked on developing a new method for computing the tomographic projections. They utilized the optical rays to compute the projections and dose simulations and computationally cast in the printing systems. They referred to the printing systems as 3D ray tracing. This new method did not require parallel rays of light as between the layers a light dose can be delivered. They demonstrated an approach that uses software for correcting the 3D non telcentricity and enhanced etendue.^21^ Pombo et al. developed paracetamol loaded tablets using tomographic volumetric printing. They used poly(ethylene glycol) diacrylate 575/700 as photoreactive monomers, lithium phenyl-2,4,6-trimethylbenzoylphosphinate (LAP) as photoinitiator, and water and PEG as nonreactive diluents. The resulted printlets were fabricated in 12 to 32 s and also showcased sustained drug release profiles. In another study, stiffness control was obtained via tomographic volumetric printing. The team used wavelength sensitive photo resins and generated high precision internal mechanical property gradients. For stiffness control, the chemistry between free radical and cationic polymerization is optimized in the solutions to obtain the stiffness control radical resolution at 300 μm.^22^ Weisgraber et al. developed a simulation known as VirtualVAM that helps in modeling tomographic VAM processes. VirtualVAM, from any tomographic VAM print is able to generate a large data set by getting better insights of reaction, diffusion, and heat reaction and helps in optimization of exposure patterns. The basic difference between VAM and VirtualVAM is that the former focuses on the production of parts via tomographically defined illumination, whereas the latter uses simulation framework that mainly focus on the model and the parameters of the whole process as shown in 2and Table 2.^23^

## Difference between VAM and other Additive manufacturing Process

In additive manufacturing, transforming digital 3D models into physical objects involves intricate layering techniques and precise processes. Initially, slicing algorithms segment the model into thin, printable cross sections, determining the layer height and orientation for each segment. This segmentation is crucial for managing the anisotropic behavior of materials during printing. A wide range of additive manufacturing (AM) technologies exists to accommodate various materials, from polymers to metal alloys, each posing unique challenges related to thermal dynamics, rheology, and postprocessing requirements.^24−26^ The two main printing techniques used in AM are point-by-point and layer-by-layer printing.

In point-by-point (1D) printing, objects are constructed sequentially, building one point at a time. This approach is utilized in several AM methods, including binder jetting, where a precisely controlled nozzle deposits a binding agent onto a powder bed, adhering particles together.^27−29^ It is also employed in material extrusion, in which a nozzle extrudes material onto the build platform in a controlled manner to form layers.^30,31^ Another example is laser sintering, where a laser selectively fuses or melts powdered material at the surface, layer by layer.^32,33^ To address the relatively slow speed of point-by-point printing, advancements have been made using multiple fabrication points. For instance, laser diode arrays have been integrated into laser sintering, enabling simultaneous sintering of multiple points and significantly enhancing throughput efficiency^34^

Layer-by-layer (2D) printing advances the process by simultaneously exposing an entire layer to an energy source simultaneously. This technique is fundamental to vat polymerization technologies, where an ultraviolet light source polymerizes the photoactive resin, solidifying an entire layer in one step.^35,36^ Digital Light Processing (DLP) units and Liquid Crystal Display (LCD) masks play key roles in shaping and modulating light exposure to accurately match the intended geometry of each layer. The layer-by-layer approach offers advantages such as faster printing times and improved precision in layer alignment. A notable advancement in this method is Continuous Liquid Interface Production (CLIP), which utilizes a continuous fabrication process rather than discrete layers, significantly speeding up production. Developed by Carbon3D, CLIP maintains a steady UV light flow and oxygen permeability control at the resin interface, leading to rapid and smoother resin curing.^37^ However, the layer-by-layer method also poses challenges such as ensuring uniform adhesion between layers and managing thermal stresses during solidification.

Volumetric additive manufacturing (VAM) represents a more complex approach, extending beyond traditional 1D and 2D methods by utilizing advanced volumetric exposure method (VEM) algorithms. These algorithms transform 3D model data into commands for energy transmitters, as illustrated in Figure 1. Unlike conventional methods that divide models into individual layers or points, VAM generates an exposure dose distribution function (EDDF) to guide the formation of the desired 3D shape within the medium. A significant advantage of this technique is its reduction in moving parts and avoidance of repetitive, lower-dimensional operations such as layer-by-layer or point-by-point printing.

Another unique feature of VAM is its ability to create objects without requiring additional material input during printing. Instead, it uses a preprepared medium activated by energy, typically light, from energy transmitters. This eliminates the need for adding more material during fabrication. In contrast, other AM methods, such as vat polymerization, involve repeatedly immersing the build platform into the medium to build layers, while material extrusion requires a continuous supply of material to construct the object.

Monovoxel printing, as outlined by Sanders et al.,^38^ involves an up-conversion process in which lower-energy photons are absorbed and re-emitted as higher-energy photons. This enables the printing of 3D voxels (essentially 3D pixels) within a three-dimensional space without adding material to the build volume. During printing, the laser is moved incrementally in smaller operations to draw the part, making it a lower-dimensional process. Although Monovoxel printing is applicable to some aspects of volumetric additive manufacturing (VAM), it employs lower-dimensional operations (1D) and does not achieve complete printing in a single step.^39^

Xolography, which uses dual-color photoinitiators, could also be seen as not entirely meeting the definition of VAM, given that the illuminated plane traversed by the Gaussian beam can be perceived as lower-dimensional (2D).^40^ Nonetheless, the rapid nature of the process and the use of superimposed light warrant its inclusion in the criteria. This method is expected to support two promising areas of future research within VAM: overprinting, where Xolography could be conceptualized as volumetric segments represented by planes, and the application of moving energy transmitters in VAM. These aspects will be discussed further in a subsequent discussion section.

With a clear definition of VAM established, the next section will present the specific criteria used for selecting and reviewing the relevant literature. A systematic approach was employed to provide a comprehensive overview of the current body of research in this field, with the aim of allowing future studies to adopt the same methodology for comparative analysis, thereby facilitating the comparison of the available literature over time.

## Materials for VAM

The choice of materials for VAM is very crucial, as the transparency and the optical density play an important role for volumetric printing. The mechanical properties, including softness and stiffness, should be balanced in the material of choice. Most commonly used materials are acrylates, epoxies, thiol–enes, sintered materials and hydrogels as mentioned in Table 3 and Table 4.

**Table 3:** Materials for VAM

Material	Description	Properties	Applications	Common Printers	Specific Bioink Names	Specific Material Names
Acrylates	Acrylates are a group of fast-curing photopolymerizable resins widely used in VAM due to their ability to solidify quickly when exposed to light.	- High resolution	- Prototypes	- SLA printers	N/A	- PEGDA (Polyethylene Glycol Diacrylate)
		- UV curable	- Biomedical devices	- DLP (Digital Light Processing)		- HEMA (Hydroxyethyl Methacrylate)
		- Low viscosity	- Electronics housings			
		- Good mechanical properties				
Hydrogels	Hydrogels are water-absorbent materials with a high degree of flexibility, often used for biomedical applications in VAM.	- Biocompatible	- Tissue engineering	- Bioprinting systems	N/A	- PVA (Polyvinyl Alcohol)
		- High water content	- Drug delivery	- Micro-SLA printers		- PEG (Polyethylene Glycol)
		- Flexible	- Wound dressing			
		- Soft mechanical properties				
Cell-laden Hydrogels	Hydrogels embedded with live cells for bioengineering applications; used in bioprinting and regenerative medicine.	- Supports cell growth	- Organ printing	- Bioprinting systems	- GelMA	- GelMA (Gelatin Methacryloyl)
		- Biodegradable	- Tissue scaffolds		- Alginate	- Fibrinogen
		- Biocompatible	- Personalized medicine		- Collagen	
		- Complex structural integration of cells			- Fibrin	
					- PEGDA	
Sintered Materials	Sintered materials are created by heating powdered metals, ceramics, or polymers just below their melting point to create a solid mass, often used for VAM in SLS.	- High strength	- Aerospace parts	- SLS (Selective Laser Sintering) printers	N/A	- Aluminum Oxide (Al2O3)
		- High temperature resistance	- Automotive components			- Titanium Alloy (Ti-6Al-4 V)
		- Porous structures	- High-temperature applications			
		- Thermally stable				
Epoxies	Epoxies are thermosetting polymers that are used in VAM for their excellent mechanical properties and chemical resistance.	- High strength	- Structural parts	- SLA printers	N/A	- Bisphenol A Epoxy Resin
		- Chemical resistance	- Coatings	- DLP printers		- Cycloaliphatic Epoxy
		- High bonding properties	- Adhesives			
		- Can be brittle at low temperatures				
Thiol–enes	Thiol–ene systems are a class of photopolymers used for their rapid curing and tunable mechanical properties, often used in specialized VAM processes.	- Rapid curing	- Biomedical devices	- SLA printers	N/A	- Trithiol (Trimethylolpropane Tris(3-mercaptopropionate))
		- Tunable flexibility and rigidity	- Microfluidic devices	- Micro-SLA printers		- Pentaerythritol Tetrakis(3-mercaptopropionate)
		- UV curable	- Electronics prototyping			
		- Low shrinkage				

**Table 4:** Materials used in the Volumetric Additive Manufacturing and its advantages

Materials	Advantages	References
Gelatin Methacryloyl	Low surface roughness, appealing for optical applications	(12)
Acrylic, Urethane and Silicone parts	Fabrication of soft elastomeric parts,	(7)
	Surgical model of soft organs	
Hydrogel based bioresin	Provided important knowledge on the ideal design requirements, and fabrication of multiscale biofactories capable of guiding tissue-specific functions.	(2)
Paracetamol loaded printlets	Fastest way of producing (7–17 s), suitability of orienting medicines at rapid speed	(20)
Silk Fibroin screws	Low concentrations of SF used, gave a better spatial structure	(28)
Gelatin Methacryloyl	Perfusable prevascularized construct with endothelial cell lining was established successfully	(4)
Acrylate and Epoxy monomers solution	Improves the geometric fidelity	(34)

### Acrylates

Acrylates are basically salts, esters and conjugate bases derived from acrylic acid, employed in VAM due to their high reactivity, adhesiveness, low cost, and tunable mechanical properties.^13^ The polymeric form of acrylates is known as polyacrylates, which consists of multiple functional domains.^22^ They polymerize rapidly in the presence of free radical in photoinitiator and the reaction halts whenever the oxygen scavenges free radicals.^23^ Because of this process, in response to light dose there is nonlinear threshold conversation that helps printing in VAM.^24^ The advantage of using acrylates for VAM is that the objects have an almost perfect balance between stiffness and softness. These materials have their limitations in cell printing as they are cytotoxic and require precise control during polymerization.^41^

Loterie et al. used dipentaerythritol pentaacrylate and 0.6 mM phenylbis to produce photosensitive resin for fabrication of hearing aids and arteries. The CT scans of artery and hearing aid was obtained and then reconstructed using a software. The optical setup was done using 6 laser diodes, which were combined to form a single beam. The light efficiency was enhanced by using rectangular aperture of the modulator that allows homogenizing the beam. The magnification and projection of the output were done using a digital micromirror device (DMD) consisting of an aspheric lens. DMD was fixed in a manner to allow diagonal tilt in the axis, giving a rotation angle of 7°. The intensity images are recorded using a camera, filtered, and down sampled so that the background noise was removed. The researchers showed that the illumination system and the resin viscosity is required to achieve maximum intensity of light and also showed the ultrafast production of constructs with precision.^7^ Boniface et al. focused on the size of objects that can be printed using a volumetric printer. They proposed on using volumetric helical additive manufacturing that will scale up the structures without magnifying the projected patterns and also not affecting the final resolution as shown in Figure 3. For the same, they used liquid pentaacrylate mixed with phenylbis (2, 4, 6-trimethylbenzoyl) phosphine oxide. A 32 mm cylindrical glass vials were used and 405 nm laser diodes combined to form a single beam with the help of a D-shaped mirror used in the optical setup. The magnification of the optical beam was done using DMD’s rectangular aperture, and the surface of the DMD is imaged by the cylindrical vial that contains the resin. The setup helped in increasing the printing voxels by a factor of 12 while having the same conventional modulating device by vertically translating the resin continuously along the light beam. This setup helped in fabricating high resolution and high-speed objects with size 3*3*6 cm. It has potential application in the field that requires rapid printing with larger dimensions such as dental aids.^27^

### Hydrogels and Cell-Laden Hydrogels

Hydrogels are an aqueous network of hydrophilic polymers and show the property of preserving the cell viability by embedding the cells and also retaining the functionality of the cells. In the biofabrication technique, the control over spatial arrangement of cells is being leveraged by the researchers to mimic the native biological systems. The newest addition to biofabrication and 3D bioprinting is volumetric bioprinting, which fabricates cell laden functional tissues within microseconds for tissue engineering and modeling.^26^ Hydrogels showcases poor mechanical strength and shrinkage during gelation and also limited cell infiltration in dense networks.^42^ One of the first biomaterials to be studied for VAM was gelatin methacryloyl (GelMA). Gelatin is formed by partial synthesis of collagen and is widely used in tissue engineering due to its biocompatibility, biodegradability, and promotion of cell adhesion and cell function. In 2000, the first report on the production and application of (meth)acryloyl modified gelatin, or GelMA, was published. By altering the GelMA prepolymer content and the extent of methacryloyl substitution, the (meth)acryloyl moieties undergo free-radical polymerization in the presence of a photoinitiator, rapidly producing a covalently cross-linked hydrogel with a mechanical profile that can be altered.^28^ Gelatin is aqueous at 37 °C and by cross-linking the optimal printability can be obtained. GelMA bioresins can be used to form organoids using volumetric bioprinting. The cells are not stressed nor sheared due to nozzle free printing. GelMA laden with mesenchymal stem cells were used to see the expression of osteoblasts and osteocyte markers for the application of bone tissue engineering.^12^ Furthermore, many research laboratories are able to obtain GelMA commercially or through synthesizing in the laboratories through clearly defined protocols. The various pathways have been developed such as endotoxin-free, medical-grade material processing pathways, which make it easier to potentially translate GelMA- based constructs into pharmaceutical and medical goods. In view of this, GelMA quickly rose to recognition as the most popular bioinks for different 3D printing methods such as extrusion based bioprinting and light-based bioprinting.^28^ Silk, is also being used as a material for volumetric printing as the mechanical properties can be tuned and the required complexity in structure can be attained. *Bombyx mori* comprises silk fibroin and silk sericin which contains different amino acids.^27^ Silk can be used directly for VAM due to development of Ru/SPS, which is a visible light photoinitiator and helps in cross-linking by forming dityrosine bonds. Using silk fibroin and silk sericin, shapes like pyramid, ring, and brainlike structures were printed volumetrically. It was printed within few tens of seconds with printability range as 2.5–5% with 0.25–1 mM of Ru/SPS.^28^

### Sintered Materials

Sintered materials possess properties that help in various technical and industrial applications like hardness, chemical resistance and inertness.^29^ The most commonly used materials include ceramics and glasses, having the properties of optical transparency and refractivity.^30^ Usually, a green body is fabricated that combines the shapeability and resistance of plastics and ceramics respectively. It has low stiffness and has been molded in the required shape. The green body is pyrolyzed and sintered at high temperature, burning all of the organic compounds present in the green body. Molten glass filament deposition and powder-based sintering have been used to freeform 3D shape in glass.^31^ Sintered materials have high processing temperatures which are incompatible with cell printing and often causes brittleness that limits the structural applications.^43^ Kollep et al. used tomographic volumetric printing to fabricate 3D ceramic parts. Polysiloxane preceramic resin and diacrylate as cross-linker were used, and the solution was poured in cylindrical vial which was photocured using light source of 405 nm wavelength. Complex shape parts were properly fabricated, and the green bodies were pyrolyzed to convert into ceramics. The components fabricated showcased strong resistance to harsh environment and also the there was no significant anisotropy observed in the shrinkage from polymer to ceramic conversion.^34^ Toombs et al. introduced a technology for fusing silica components and fabricating three- dimensional microfluidics. Microscale computed axial lithography focuses on providing light dose to the material that is outside the target geometry. They utilized binder matrix and silica nanocomposites to print the construct. Post printing, cleaning was done with ethanol, and then the green body was subjected to thermal treatment where it underwent debinding and sintering. Debinding helped in removing the binder matrix, and sintering helped in fusing the silica nanoparticles together to form transparent dense glass part. The advantage of this system was fabrication of sizes as minimum as 20 and 50 μm in the case of polymer and fused silica respectively. The objects had low surface roughness, excellent geometric freedom with high fracture strength and transparency.^18^

### Epoxies

Epoxies are a family of thermoset polymers that are used as materials for dental molding, surface coaters, and 3D printing. They showcase high tensile properties, along with compressive strength and thermal stability. The chemical composition of epoxies comprises of epoxide and hydroxyl groups and are cross-linked to polymerize into rigid three-dimensional lattices. Polymerization may work by direct reaction using catalyzed homopolymerization and via use of a chemical that acts as a coupling agent helping in polymerizing the epoxide groups. Epoxies have high mechanical properties but showcases poor cytocompatibility and also cytotoxicity which can limit the biological systems.^44^ Larsen et al., used monomer solution of polyethylene glycol diacrylate and epoxy to fabricate multimaterial structures which when exposed to blue light forms a soft hydrogel and when exposed to UV light forms hard solid.^35^ In the study they also used a panel of photoinitiators such as camphorquinone, triarylsulfonium hexafluoroantimonate salts based cationic photoinitiators. This was the first reported study of using acrylate and epoxy together and selectively polymerizing it with blue light. The characterization tests focused on curing rate, stiffness and the swelling and it was observed that the multimaterial have potential application in the field of tissue engineering due to its freely tunable mechanical properties.^35^ Wang et al. used a mixture of epoxy and mixed acrylate to have stiffness control over the fabricated object. Free radical and cationic polymerization orthogonal chemistry was utilized for the same. Dual color tomographic volumetric printing was used where visible light and UV light were utilized to understand the chemistry where the former excites radical photoinitiator in acrylates whereas the latter produce Brønsted acid by exciting the cationic photoinitiator. The lattice formed by the two materials that are not covalently linked at the same time helps in procuring a multimaterial mix that has tunable properties.^36^

### Thiol–enes

Thiol–enes is a chemical reaction that takes place between a thiol and an alkene and forms a product known as a thioether. This reaction is a part of click chemistry and due to presence of vast thiols, the family of thiol–enes is huge.^37^ The networks formed by the reaction between two groups are more uniform in comparison to acrylates, and they also have high refractive index and optical clarity. When it comes to the utilization of thiol–enes for VAM, it has its own set of advantages and disadvantages. They are formed by rapid reaction that goes hand in hand with VAM and provide higher biocompatibility and biodegradability that may make them suitable for VAM bioprinting. The disadvantage of working with thiol–enes for VAM is that they are insensitive to oxygen, making it a challenging as the gelation post printing is controlled by the inhibitory mechanism and also the UV cross-linking may cause cytotoxicity and limit the mechanical durability.^45^ For inducing the threshold in thiol–enes, 2,2,6,6-tetramethyl-1-piperidinyloxy (TEMPO) was used that acted as a radical scavenger.^19^ The development of photo-cross-linkable poly(ε-caprolactone) networks using orthogonal thiol–ene chemistry as an alternative to acrylate cross-linking was studied. These networks offer improved mechanical properties compared to acrylate-cross-linked materials, addressing their brittleness issue.^46^ Tunability was achieved by adjusting the molar mass between cross-links (Mc), allowing for control over thermal properties, mechanical strength, and degradability. Additionally, the materials exhibit outstanding volumetric printability, enabling the creation of structures with the smallest features reported for thiol–ene systems via volumetric 3D printing. This advancement expands possibilities for precise and intricate fabrication in 3D printing applications. The biocompatibility of the volumetrically 3D-printed materials was studied through *in vitro* and *in vivo* characterization of 3D-printed constructs. This aspect was critical for potential biomedical applications, indicating the suitability of these materials for use in patient-specific implants. Overall, the combination of mechanical stability, tunability, biocompatibility, and rapid fabrication by volumetric 3D printing offers a promising approach for the bedside manufacturing of biodegradable patient-specific implants. This research represents a significant advancement in biomaterials and additive manufacturing, with potential implications for personalized medicine and tissue engineering.^46^

## Applications of VAM

**Figure 10:**
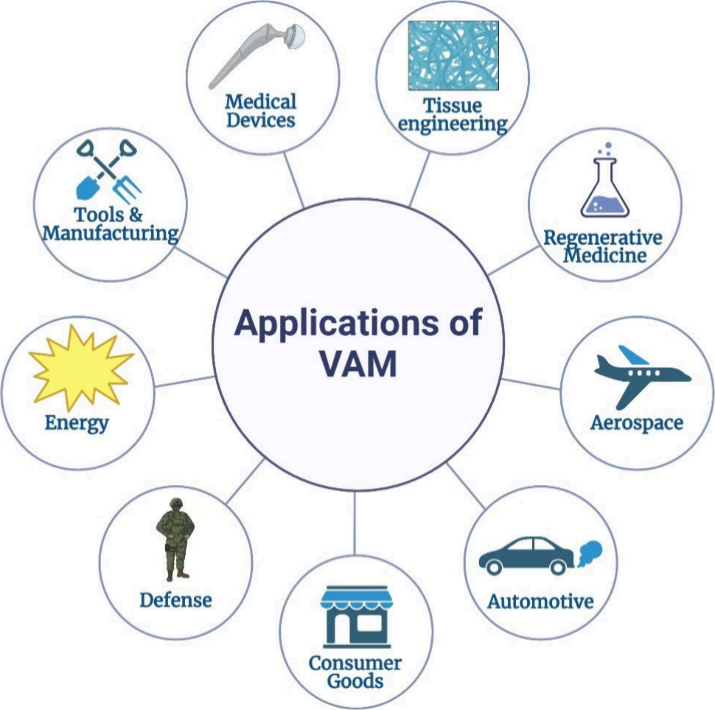
Applications of VAM.

The versatility of VAM holds a diverse array of applications, exemplified by influential works in the field, some of those have been highlighted in this review (Table 5 & Figure 10). Kelly et al. introduced Computed Axial lithography (CAL), a novel technique facilitating the volumetric synthesis of complex geometries through photopolymerization, accommodating both high-viscosity solids and fluids. Their methodology promoted the fabrication of 3D structures close to those of existing solid components. Interestingly, this approach demonstrated the scalability for larger print volumes and significantly outperforms traditional layer by layer methods in terms of speed across a wider range of operational conditions. The system architecture employed in CAL fabrication offers significant advantages, particularly when handling materials with low elastic modulus, typically ranging from 1 to 10 kilopascals, as these are commonly utilized in various soft tissue modeling and bioprinting applications. This was exemplified through their experimentation with 3D patterning of the gelatin methacrylate (GelMA) hydrogel material. The conventional layer by layer printing of these material would present challenges due to the forces exerted on incomplete structures during the printing process.^14^ Loterie et al., showcased high-resolution volumetric production of centimeter-scale acrylic and silicone parts using feedback-enhanced tomographic reconstruction, achieving rapid printing of centimeter-scale soft silicon arteries in less than 30 s.^7^ VAM has proven to be a promising method for microneedle fabrication, as highlighted by Makvandi.^40^

**Table 5:** Application of VAM

Area^a^	Description	Common Materials
A	Used to produce lightweight, high-strength components such as engine parts, brackets, and structural elements. Reduces material waste and enables the creation of complex geometries that enhance performance in aircraft and spacecraft. Common materials include titanium, aluminum, and high-temperature alloys.	Titanium, Aluminum, High-Temperature Alloys
AT	Enables the production of lightweight automotive parts, reducing fuel consumption and improving vehicle performance. Additive manufacturing is used to create components like customized dashboards, structural parts, and engine components. It also supports rapid prototyping for design iteration.	Steel, Aluminum, Polymers
M	Facilitates the production of patient-specific implants, prosthetics, dental devices, and surgical tools. Additive manufacturing allows for customization and biocompatibility with the patient’s anatomy, improving the effectiveness of treatments. Materials include biocompatible metals, polymers, and ceramics.	Biocompatible Metals (Titanium, Cobalt-Chrome), Polymers, Ceramics
CG	Used to produce customized consumer goods such as footwear, eyewear, electronics, and household items. Additive manufacturing allows for rapid production of complex designs and personal customization, while minimizing waste. Common materials include polymers and elastomers.	Polymers, Elastomers
C	Additive manufacturing is employed in construction to produce modular building components, structural elements, and even entire buildings. The technology allows for reduced material waste, faster construction times, and the ability to create more sustainable, customized structures.	Concrete, Polymers, Recycled Materials
D	Volume additive manufacturing is used to produce lightweight, high-performance components for defense applications, such as unmanned vehicles, weaponry, and protective gear. The ability to quickly iterate designs and produce low-volume, mission-critical parts is a significant advantage.	Composites, High-Strength Alloys
E	In the energy sector, additive manufacturing is used to create parts for turbines, fuel cells, and oil and gas equipment. The technology enables the production of highly durable components with improved efficiency, particularly in harsh operating environments.	Nickel-Based Alloys, Steel, Polymers
T	Widely used for rapid tooling, mold making, and prototyping across various industries. Volume additive manufacturing enables faster design iteration and cost-effective production of specialized tools, molds, and prototypes, reducing lead times and development costs.	Polymers, Metals, Tool Steels

a A: Aerospace, AT: Automotive, CG:Consumer goods, C:Construction, D: Defense, E;Energy, T: Tools and Manufacturing

In tissue engineering, Bernal et al. utilized hydrogel-based bioresin in VAM to construct a large living tissue, specifically a human auricle, by using gelatin methacryloyl. They successfully fabricated trabecular architecture and the convoluted, interconnected porous network using the VAM approach. Additionally, they developed fully perfusable hollow channels with an inner diameter of 200 μm. Furthermore, a meniscus-shaped construct was bioprinted to assess the synthesis of matrix and neo-tissue formation. A fluidic valve for cardiac valve prosthesis was manufactured, confirming that the VAM offers the design of more complex, hollow, and free-flowing structures without any support system. All models were printed in the same duration of time and without any support structures. They then proceeded to demonstrate that the creation of the living tissue constructs did not affect the survival of the cell. The volumetric-based 3D printing exhibited promising volume accuracy. Additionally, they successfully combined VAM with organoids, showcasing the printing of epithelial liver-derived organoids that preserved morphology and enhanced viability postprinting. In addition to this they also proved that the nozzle-free method does not pose any risk of stress induced cell damage that could alter the function of biological construct.^2^

Gehlen et al. introduced new strategies for the rapid fabrication of heterocellular bone-like tissues using ultrafast VAM. The optimized formulation of photocurable gelatin methacryloyl bioresins was meticulously chosen, having been identified for its optimal printability, mechanical properties, and a favorable environment for osteogenic differentiation of human mesenchymal stem cells (hMSCs). They established a 3D endothelial coculture to test if the heterocellular interactions may enhance the osteogenic differentiation in the printed environment. For the applications of vascularized tissue engineering, a perfusable cell laden tissue construct was demonstrated with imprinted channel structures.^6^

Pombo et al. employed VAM technology to develop 3D printed printlets containing paracetamol. They exhibited efficacy in consistently fabricating personalized medicines across six distinct formulations. The successful fabrication of paracetamol-loaded torus shaped printlets was achieved within a time frame ranging from 7 to 17 s, representing the fastest method for producing printlets via 3D printing. The selection of the torus shape was primarily motivated by its superior surface area to volume ratio compared to a disc, which resulted in an accessibility score in similar investigations.^47^

Xie et al. employed silk fibroin and silk sericin as bioinks for volumetric 3D bioprinting. Optimization of the silk fibroin and silk sericin was carried out after the assessment of their printability at different concentrations and various photoinitiator ratios. Their findings revealed that the lower concentration of silk fibroin and silk sericin inks led to better mechanical properties, unlike those in the extrusion methods. Human organ like structures including brain, ear, tooth, and kidney-like constructs were produced successfully. They investigated the printing resolutions of silk fibroin and silk sericin involved in the fabrication of diverse structures. Thus, we deduced that the resolution of printing depended on the concentration of the silk. Prolonged printing times were found to decrease the resolution and induce surface overcross-linking. Moreover, the cell-laden bioink demonstrated a favorable cytocompatibility. This study also emphasized the bone formation potential of VAM printed silk fibroin screws, featuring the double cross-linked network. Their work expanded the bioink library for VAM, offering avenues for rapid VAM of silk- based materials across various biomedical applications.^30^ There still exist many difficulties with 3D printing biological cells and tissue. Hence volumetric bioprinting, when combined with certain gels, enables the 3D printing of cells in a matter of seconds. In the traditional 3D bioprinting techniques, the cells cannot be manipulated or positioned precisely. The gels also cannot be altered to allow for cell growth, development, and specialization of the cells. To ensure the survival and growth of the 3D printed cells, extra care. Additionally, the ability of these cells to proliferate and communicate migrate is essential. Numerous cell kinds can be deposited in large quantities through extrusion 3D printing; nevertheless, these cells might undergo mechanical stress and the whole process in itself would be time-consuming. Even if volumetric bioprinting does not possess such drawbacks, the selection of the appropriate material would pose a slight challenge. Thus, materials such as soft hydrogels could be used as they would allow the communication and self-organization of the cells. The scientists at UMC Utrecht have put forth various innovations in volumetric bioprinting. They have created functional regions in 3D printed cells, they have used granular gels for the optimization of 3D bioprinted cells, and they have used the combination of volumetric bioprinting with melt electrowriting to 3D print blood vessels.^48^ They concluded that the final 3D printed structures were frequently fragile because volumetric bioprinting uses cell-friendly gels. When creating blood veins, which must endure extreme pressure and bending, this presents challenges. In order to produce stronger and more resilient structures, the researchers integrated volumetric bioprinting with melt-electrowiring. A Team at the University of Melbourne developed this new method that uses a controlled light and a limited air–liquid interface and resolution, fast, support-free bioprinted structures, opening up new avenues for the creation of intricate, cell-rich 3D structures. Through this method, large scale, complex bioprinted structures such as artificial organs, vascular networks, and multimaterial constructs. A sophisticated kidney-like hydrogel structure loaded with human embryonic kidney cells was created in one recent experiment. The procedure produced viable cell structures. Its low-intensity light levels and use of biologically safe, cell compatible materials protect cell viability and reduce cytotoxicity, both of which are essential for creating functional tissue models that mimic the behavior of actual biological tissues. The researchers have developed the paracetamol-loaded tablets within 17 s which is a lot quicker than the current techniques used to print medications in various clinical and research contexts. They were called printlets, which were in torus shape. The researchers claim that the quickest way to produce customized tablets using 3D printing to date, validates the viability of volumetric 3D printing for quickly generating medications.^19^ Although the commercialization of 3D printed medications is making headway, there are still obstacles in the way of fully transferring the technology from the lab to clinical settings. Specifically, throughput issues prevent current 3D printing methods from scaling up to produce the billions of tablets needed annually for commercial manufacturing. According to the scientists, this is the first time drug-loaded tablets have been produced using volumetric printing, and it drastically reduced the production time to a few seconds. The researchers were able to adjust the medication release rates of the printed tablets to the required speed by testing six distinct resin compositions, each of which contained paracetamol, a cross-linking monomer, and a photoinitiator.^49^

The primary applications of VAM encompass optics, dentistry, audiology, bioengineering, microfluidics, and prototyping. For the creation of 3D constructs containing cells at a centimeter scale, they require quick and scalable technologies. Although the products are still in their initial stages of development, VAM exhibits great promise. Redily3D, a startup, has introduced a VAM printer known as Tomolite, a contactless tomographic illumination technology that ensures a safe environment for the bioinks. Additionally, Redily3D is in collaboration on a project aiming to develop a living pancreas model to advance diabetes research.^50^

In the field of dentistry, volumetric 3D printers are utilized for the development of dental implants, crowns and bridges, surgical guides, and anatomical replicates and models. Furthermore, the application of volumetric 3D printing extends to maxillofacial dentistry, where implants can be manufactured by using this technology. A start-up, Vitro3D, is dedicated to advancing the dental aligner market, which remains in its initial stages of development. VAM is assisting in creating patient-specific and highly efficient hearing aid devices.^51^ (Clinical trials and patents)

## Future Perspectives of VAM

In recent years, VAM has garnered significant attention due to its notable features, including enhanced printing speed, supportless printing modalities, and favorable outcomes in maintaining high cell viability. Despite this progress, ongoing scientific efforts persist in refining various aspects of the printing process, such as resolution optimization and exploration of diverse bioinks.^52^ Looking ahead, the realization of multicellular and multilayered printing techniques is anticipated. Moreover. the development of alternative types of VAM based bioinks warrants consideration. Presently, the major limitation of 3D volumetric printing is the constrained build size of volumetric 3D printers, primarily due to the necessity for adequate light penetration to facilitate effective resin solidification throughout the volume. Another one of the major drawbacks of the VAM is that only transparent resins can be used as it ensures the minimal diffraction and light dose dependent from penetration depth.^1^ Various approaches are being explored to address these limitations. For instance, Regehly et al., proposed an alternative volumetric 3D printing strategy known as Xolography.^53^ This study employs photo switchable photoinitiators reaching to intersecting light beams of different wavelengths. This technique offers a resolution ten times higher than computed axial lithography (CAL) and increased volume throughput compared to two-photon polymerization (TPP).^54^

It is noteworthy that volumetric printing is primarily feasible for 3D objects on a scale per centimeter. Overall, there has been a remarkable development of biodegradable medical devices or implants in recent years. As there is an increasing complexity of medical devices which poses as a significant challenge, but is also a factor of motivation for research in the areas of pharmaceutical science, human medicine, material science, chemistry, mechanical engineering, and veterinary medicine. The numerous advancements in 3D printing techniques encompassing innovations in materials and high precision 3D printing techniques are quite notable. VAM 3D printing may bring about an innovative manufacturing of biodegradable medical implants and devices and would play a very important role in personalized medicine.^1^

## Regulations Associated with VAM

Volumetric Additive Manufacturing (VAM) is an upcoming technology in the field of 3D printing that can revolutionize manufacturing processes by expeditious production of large objects with complex geometrical shapes. However, like any other turbulent technology, VAM encounters challenges and regulatory considerations which should be addressed before its widespread application^55^ as shown in Table 6. Some of the regulatory issues with VAM are enlisted as follows

**Table 6:** Safety Regulations for VAM

Safety Aspect	Description	Key Standards/Regulations
Machine Safety	Ensures that additive manufacturing equipment such as lasers and electron beams are properly guarded and maintained. Regular calibration and safety checks are required to prevent accidents. Compliance with ISO 12100 and OSHA standards for machine safety is critical.	ISO 12100, OSHA (29 CFR 1910)
Fire and Explosion Prevention	Addresses the risk of fire and explosion, particularly when handling combustible powders like aluminum or titanium. Compliance with NFPA guidelines for storage and handling of combustible materials is essential, as is grounding and preventing static build-up.	NFPA 484, NFPA 652 (Combustible Dust), OSHA (29 CFR 1910.307)
Worker Health and Safety	Focuses on worker exposure to hazardous substances, fumes, and high levels of noise during the manufacturing process. OSHA standards mandate the use of engineering controls and personal protective equipment to mitigate risks.	OSHA (29 CFR 1910.1000), NIOSH (National Institute for Occupational Safety and Health)
Hazardous Material Handling	Ensures that hazardous materials, including metal powders and resins, are safely stored, handled, and disposed of to avoid contamination and environmental damage. Compliance with OSHA’s Hazard Communication Standards and NFPA guidelines is required.	OSHA Hazard Communication Standard (HCS), NFPA 400
Personal Protective Equipment (PPE)	Mandates the use of appropriate PPE such as gloves, protective eyewear, and respirators to prevent worker exposure to harmful substances or high temperatures. PPE standards align with OSHA regulations for occupational safety.	OSHA (29 CFR 1910 Subpart I)
Ventilation and Fume Control	Ensures that facilities have proper ventilation systems in place to control the release of fumes and particulate matter during the printing process. This is essential for meeting air quality standards and protecting worker health.	OSHA (29 CFR 1910.94), ASHRAE (American Society of Heating, Refrigerating, and Air-Conditioning Engineers)
Emergency Protocols	Establishes protocols for responding to emergencies, such as fires, chemical spills, or equipment malfunctions. Proper training and regular drills are required to comply with OSHA standards and local fire safety regulations.	OSHA (29 CFR 1910 Subpart E), NFPA 101 (Life Safety Code)

### Safety Regulations

Safety regulations in VAM are required to protect users and workers from potential hazards caused by the technology. VAM processes often make use of light sources with high intensity and high-power lasers which present a risk of exposure to laser radiation, elevated temperatures and chemical exposure for workers.^54^ Regulatory bodies cause imposition of stringent safety measure requirement on VAM operations and equipment’s, like interlocks, machine guarding, ventilation systems and protection equipment’s etc.^55−65^ Compliance in conjunction with safety regulations is necessary to inhibit these risks and ensure the safety of personnel involved in VAM processes, while also ensuring trust in the adoption of technology and integrating it into various industries. Additionally, industry and research collaboration are necessary for stakeholders and regulatory agencies to tackle emerging safety issues and continuously improvement in safety standards in VAM.^66−71^

### Material Regulations

Material regulations relating to VAM become necessary for safety, compliance, and care for environmental sustainability in the use of raw materials in very many industries.^72−75^ VAM employs a wide array of materials-metal, polymers, ceramics, and composites-which depend on specific regulations according to their application and potential hazard.^76−81^ Such frameworks are the ones that provide a backdrop for regulating the safe use, handling, and disposal of chemicals and materials involved in additive manufacturing processes: e.g., REACH in Europe and TSCA in the U.S. And both of these laws require the manufacturer to assess the environmental and health risks associated with using particular materials, especially those which may release harmful substances during printing or postprocessing. Certain metals are prone to fire or explosion, such as titanium or aluminum powders, and best practices would be followed, perhaps guided by third-party groups like the National Fire Protection Association.^82−84^ Similarly, toxic or hazardous substances in the manufacturing process may be limited by regulations especially for industries related to medical devices, where even standards such as ISO 10993 have to be complied with in the category of biocompatibility in order to make the material safe for use in implants or other medical tools. In addition to those mentioned above, sustainability and recyclability issues are increasingly significant in material regulations, with the aim of reducing or fully excluding waste products with a minimal footprint on the environment. Companies operating within the domain of VAM^85−87^ are encouraged-and, in some cases, bound-by sustainable practices such as powder recycling or incorporation of biobased materials to meet environmental standards such as ISO 14001. Thus, compliance with materials regulations also relates to the assurance of quality and certification within highly regulated industries, such as aerospace and automotive, where the standards for material performance are really high, usually at AS9100 and ISO/TS 16949, accordingly.^88−90^ In these industries, traceability of materials is vital, and often, manufacture has to document the whole supply chain of materials to make sure that all components in a particular product will meet safety and performance criteria. Altogether, material regulations involving VAM are all-inclusive: covering health and safety, environmental impact, and industrial standards.^91,92^ It follows that compliance with such regulations not only helps a company avoid running afoul of government agencies but also ensures a superior quality and sustainability profile and hence enhances the market competitiveness for the product or service provided.

### Intellectual Property Protection

Due to innovative nature of the technology itself along with a possibility to replicate complex design and processes, IP protection is one of the key factors in VAM. The digital models involved in VAM, such as CAD files, can be shared and modified with ease, therefore the risks of unauthorized reproduction or counterfeiting of proprietary designs and technologies go up.^93−96^ The protection of IP will necessitate the need for these companies to implement a few strategies on how to patent their inventions, processes, and designs for inventing unique innovations. This can pertain to the different methods that exist in the additive manufacturing process or the materials that have been developed for particular applications. The digital files and design blueprints may be protected with copyrights, while the trademark protects the branding elements adopted for the products. They are equally useful in the protection of confidential techniques or material formulations used in manufacturing, provided strict confidentiality measures are enacted by companies. Besides the protection methods of traditional IP, digital files need to be secured through encryption and access control in order to avoid unauthorized dissemination or theft during external collaboration or cross-border transfer in files. While additive manufacturing is genuinely globally undertaken, a leap in complexity concerning the use of international IP laws to provide protection for their innovations across multiple jurisdictions is required. Moreover, the increasing use of 3D scanning and reverse engineering in value-added manufacturing can further complicate matters, since unauthorized people are able to make copies of existing products without permission. This is a risk that companies can reduce by using digital watermarks, blockchain, or other methods of digital rights management to monitor their designs’ usage and distribution. With the continuous evolution of the VAM landscape, robust IP protection strategies will be required to preserve competitive advantage, foster innovation, and avoid unauthorized exploitation of additive manufacturing technologies.^97^

### Quality Control and Standards

Quality control and adherence to standards are vital in the course of VAM to ensure that the final product can meet specifications and performance criteria. VAM involves a rather sophisticated layered deposition of material; such processes require a similar guarantee of quality within large production volumes. International standards abound;^98,99^ for example, ISO/ASTM 52900 deals with additive manufacturing processes, materials, and products. The main problem with VAM is the variation in material properties, surface finish, and mechanical strength arising due to factors such as machine calibration, material quality, and processing conditions. It is very vital that manufacturers at all junctures of the production cycle adopt tight quality control measures right from the real-time monitoring of critical process parameters, including temperature, speed, and laser power. Nondestructive testing methods, such as ultrasonic testing, X-ray imaging, and surface scanning, are the usual types of inspections for any defects in printed components; such defects can be in the form of voids, cracks, or incomplete layers without part destruction. Material certification is another aspect in the sphere of quality control when high-performance alloys, polymers, or biocompatible materials come into play or are in use in sensitive industries like aerospace or healthcare. In addition to the property standardization of raw materials, VAM companies also have a higher demand for batch-to-batch consistency in material properties such as tensile strength, hardness, thermal conductivity, and many more.^100,101^ Additionally, there are heat treatment, surface finishing, and dimensional verification postprocessing specifications that maintain product quality. Very often, these processes are part of strict controls under industry standards, such as AS9100 for space applications and ISO 13485 for medical applications. Other important aspects of VAM for quality control include traceability during the manufacturing process. The source and composition of the materials used; the machine settings, and processing conditions for each part produced have to be documented by the manufacturer. On the other hand, such traceability allows a far more effective quality management, and it helps to identify the source of a specific defect or failure only in the finished product. The processes of validation and certification are crucial with respect to establishing whether the VAM processes comply with the regulatory requirements, especially in very sensitive safety applications. For instance, in industries like medical and aerospace, additive manufacturing normally has to be validated from the point of view of making a part consistently within tight tolerances, which ensures that all the safety standards are met. First, one has to run multiple test builds and then analyze the results, and then one can even certify a process based upon the performance of the parts produced. This normally means that regulatory bodies, the FDA or EASA, depending on the location, will require very strong validation of the manufacturing process with subsequent capability to be repeatably reproduced. Finally, continuous improvement represents another important characteristic of VAM quality control, with new technological advances and a new generation of materials coming forward regularly.^102−104^ This would be achieved by the implementation of an appropriate QMS that caters for the demands of constant monitoring, feedback mobilization toward optimization, and an endless struggle to meet the expected standards set by a continuously changing industrial context. With regard to such a state of affairs, strict quality control, based on established standards, allows companies operating in VAM to ensure the reliability of their products and customer satisfaction and to comply with legal and other regulatory frameworks while minimizing defects and excess variability in such high-volume manufacturing processes.

### Environmental Regulations

Environmental regulations for VAM ensure that the output produced does not harm the environment, while making certain that the processes of production are according to national and international environmental standards. Since most of the technologies generally involve VAM and materials like polymers, metals, and other chemicals that could lead to hazardous wastes or gases being emitted, various aspects of production, waste disposal, and use of energy are strictly directed.^105−107^ The main concerns relate to the management of volatile organic compounds, particulate matter, and fumes from printing through postprocessing. These emissions pose serious health and environmental risks. The facilities also have to abide by the regulations related to the Clean Air Act in the U.S. and other similar frameworks of other nations, which may stipulate air quality controls, fume extraction systems, and regular emissions levels monitoring.^108^ The other important regulatory focus is waste management, in particular, the disposal of unused or contaminated powders, failed prints, and postprocessing chemicals.^109^ The facilities dealing with hazardous materials must, therefore, use strict protocols with regard to the identification, proper storage, and, finally, disposal of such substances via RCRA in the United States or other environmental laws with respect to country specifics. These regulations also require the manufacturers to record and report on the type and quantity of hazardous waste being generated by those industries and engender good practices for its management or recycling to avoid environmental hazards.^110^ Furthermore, energy consumption by the VAM technologies has also become one of the critical issues: large-scale 3D printing processes, in particular using lasers or electron beams, can be quite energy-intensive. Apart from that, the regulatory bodies are putting immense pressure on energy-efficient practices by insisting that companies adopt renewable sources of energy, reduce carbon footprint, and increase energy efficiency for the manufacturing systems. Besides, the VAM facilities have to be sensitive to raw material extraction and sourcing from an ecological perspective. Most of the additive manufacturing materials, such as metals and some polymers, involve an energy-intensive production process, which contributes to greenhouse gas emission. This, in turn, encourages, sometimes obliges, companies to attain supplies in a sustainable manner and to be certified to standards such as ISO 14001 on Environmental management that outlines the Organization’s requirements for minimizing its impact on the environment. An Assessment on the life cycle of a product, or LCA, where one assesses a product with regard to environmental effects concerning raw material supply to production, usage, and/or deposition at the end of the useful life is also encouraged for companies. These assessments could also unveil waste reduction opportunities, efficiency in the use of materials, and enhancement in recycling. Finally, many environmental regulations require VAM facilities to perform periodic environmental audits and reports with the intent of ensuring that legal and corporate sustainability expectations are being met. In part, this may be due to their inclusion in broader CSR programs through which a company would like to show its interest in environmental stewardship. VAM facilities, by adhering to environmental legislation, will avoid fines and possible legal jeopardies, thus improving their reputation as responsible manufacturers with sustainable enterprises in the global market.^111−113^

### Export Controls

Export controls for volume additive manufacturing (VAM) are crucial due to the potential dual-use nature of the technology, which means that while additive manufacturing can be used for civilian applications, it can also be used for military purposes.^114,115^ These regulations aim to prevent the unauthorized export of sensitive technologies, materials, and digital data that could potentially be exploited for harmful or unlawful activities. Export controls are especially important in industries such as aerospace, defense, and medical devices, where sensitive intellectual property and materials are often involved.

#### Key Aspects of Export Controls for VAM

##### Dual-Use Technologies and ITAR Compliance

Most additive manufacturing technologies, particularly in dealing with metals and advanced materials, fall under dual-use groupings of export controls, such as those subjected to the US International Traffic in Arms Regulations (ITAR) and the Export Administration Regulations (EAR). ITAR regulates the export of defense-related technologies, while EAR controls items that can serve both civilian and military use. Companies involved in VAM must be compliant with ITAR and EAR to prevent unauthorized exports of technologies or data that may be used in the development of weapons or other defense applications.^116−118^ This also involves disallowing the transfer or exportation of sensitive design, materials, and equipment to any restricted country or individual without proper licenses.

##### Digital Data and CAD File Exports

One of the most difficult nuances of VAM export controls is the digital nature of the technology. In addition, CAD files or other 3D printing digital blueprints can easily cross borders via email or cloud storage and may be viewed, under some regimes, as an “export.″. Organizations should, therefore, be very aware of who has access to such digital files that can be classified as sensitive and must ensure that such files are not shared with foreign nationals or entities without appropriate licensing.^119,120^ Encryption of digital files and ways of secure transmission hence become indispensable for compliance with the export regulations in order to avoid any unauthorized transfer of sensitive information. In addition, companies may be required to obtain an export license to distribute specific files to foreign recipients or collaborators.

##### Material and Equipment Controls

These controls also extend to the materials involved, such as advanced metals, composites, and powders, that could be of strategic or military use. Certain metals, like strategic titanium and nickel alloys, along with high-performance polymers are controlled under either EAR or ITAR for export. Additionally, export controls can be applied to VAM equipment based on the sensitivity of an item, such as high-powered lasers or electron beam machines, or industrial-scale 3D printers that are considered dual-use items.^121,122^ Companies will need to verify that they are not exporting restricted equipment to forbidden destinations without proper clearance from relevant government agencies.

##### Restricted Parties and Sanctioned Countries

VAM companies must be aware of the restricted parties and countries that they are prohibited from exporting. The U.S. Department of Commerce’s Bureau of Industry and Security (BIS) maintains a list of entities and countries that are subject to export restrictions, including embargoed nations or those under trade sanctions.

Businesses must screen potential customers, suppliers, and partners to ensure that they are not engaging in transactions with entities on restricted lists. Failure to comply with these regulations can lead to significant penalties, including fines and the loss of export privileges.

##### Export Licenses and Compliance Programs

These controls also extend to the materials involved, such as advanced metals, composites, and powders that could be of strategic or military use. Certain metals-like strategic titanium and nickel alloys-along with high-performance polymers, are controlled under either EAR or ITAR for export. Additionally, export controls can be applied to VAM equipment based on the sensitivity of an item, such as high-powered lasers or electron beam machines or industrial-scale 3D printers that are considered dual-use items. Companies will need to verify that they are not exporting restricted equipment to forbidden destinations without proper clearance from the relevant government agencies.

##### Emerging Technologies and Evolving Regulations

The VAM regulatory environment is rapidly changing, as governments increasingly recognize the potential impact that additive manufacture may have on national security. Technologies such as bioprinting, metal additive manufacturing, and multimaterial printing are under particular review.^123−125^ Additional controls on emerging technologies such as these may well be further developed as these governments continue to assess their potential risks. Nanoparticles, airborne particles, and volatile organic compounds (VOCs) play important roles as enablers and considerations for innovative processes and for material and environmental safety, respectively.

The high surface area to volume ratio along with tunable properties of nanoparticles helps in exploring the functionality and performance of the printed parts, and with VAM the optical and mechanical properties of the materials are enhanced. Gold, silver, and carbon-based nanoparticles are used as they serve as photoinitiators to improve the polymer stability and resolution and enable multifunctionality such as electrical conductivity and antimicrobial properties. While it enables advancements in VAM, it poses challenges such as potential toxicity and environmental impact. The small size of the nanoparticles can penetrate biological barriers and can raise concerns about occupational health. During VAM the emission of airborne particles is a critical consideration due to impacts on environmental safety and human health. The particle size ranges from nanometers to micrometers and originates in the process of material degradation and thermal processes. Similarly, VOCs are byproducts of VAM processes and are released during printing and postcuring stages. They affect air quality and health risks due to toxicity and carcinogenicity. Emerging research are designing VOC free or low materials to minimize the exposure.^126−130^

Companies may consider trying to remain up-to-date on the latest changes in regulations by closely working with the attorney or relevant compliance departments to ensure that they are found to be current and compliant with any altering requirements in export controls.

Control exports in volume additive manufacturing play a significant role in preventing sensitive technologies, materials, and digital assets from military or illicit use. Compliance with ITAR and EAR-like regulations becomes topical when companies involved in VAM work for defense, aerospace, or other high-tech industries. These regulations dictate not only physical exportations of machines and materials but also the transmission of digital files, making tight control over both hardware and information a matter of great urgency. In order not to incur severe penalties and legal risks, companies will have to establish strong compliance programs,^131,132^ obtain necessary export licenses, and remain aware of continuously changing regulations in this fast-advancing field.

#### Data Security and Privacy

In this regard, security and privacy of data are of utmost importance in VAM, as industry people heavily depend on digital files and advanced manufacturing processes. Any business, in this line, has dire needs to protect the little things such as IP, sensitive production data, and the integrity of digital design.^133,134^ Most importantly, these are those industries where confidentiality may be paramount, such as in aerospace, defense, or medical devices, among others.

VAM, by nature, employs CAD files, 3D models, and other proprietary digital assets. Systems often move down these different stages of fabrication: design, simulation, production, and postprocessing. Breach of access to such files could mean intellectual property theft, counterfeiting, or even sabotage, which are capable of bringing a company to its financial and reputational ruin. Thus, cybersecurity must be robust enough to protect sensitive assets.

Among the key problems of data security in VAM, it is relevant to single out the integrity and protection of digital design files throughout their entire life cycle. That is, using encryption technologies protecting files both during transmission and storage and mechanisms of authentication and access control allow access only to authorized personnel. In this respect, companies have to develop and apply protocols of end-to-end encryption and check access logs regularly for any breaches that may happen.

Cybersecurity measures have to be developed in such a way that segregates these kinds of risks from the IoT and connectivity of VAM equipment. In an additive manufacturing facility, machines and systems are often networked to observe operational efficiencies, which again are potential victims of cyberattacks. The industries should implement an industry-standard cybersecurity framework, such as the NIST Cybersecurity Framework, that can help them protect their manufacturing networks from intrusion and thereby prevent malicious activities that might disrupt production or even the disclosure of sensitive data. Besides data security, there is an important role of privacy regulations while dealing with personal data, specifically in industries like health care. Additive manufacturing of medical devices or patient-specific implants relies on the processing of patient data, which in turn is subject to privacy regulations such as the General Data Protection Regulation in Europe or the Health Insurance Portability and Accountability Act in the U.S. It thus requires organizations to apply appropriate measures to anonymize personal data, store them, and process them in compliance with relevant privacy laws so that regulatory fines and loss of stakeholder confidence can be avoided. Second, intellectual property protection is an evolving concern within Virtual Augmented Makeup, in which the nearly effortless replication of digital designs carries more significant risks related to theft and counterfeiting of the IP. Companies can adopt digital watermarking approaches or blockchain-based solutions for tracing and verifying the origin of digital files that have been used in an additive manufacturing process. Consequently, manufacturers will be able to track the origin and ownership of the design files and ensure that unauthorized copies have not been produced. In a nutshell, data security and privacy in volume additive manufacturing have to be dealt with at length by strong encryption, access control, cybersecurity measures, and compliance with the regulations on privacy. If focused on these aspects, companies will be able to shield their digital assets and IP and build confidence among clients and partners in a world where the ability to manufacture more will become increasingly connected and digitized.

#### Workplace Regulations

The application of VAM is found in industries related to aerospace, automotive, and medical devices, among many others. Various workplace regulations govern the process in terms of safety, taking care of environmental concerns, and for efficiency in operations. OHS mainly deals with the safety of equipment through strict machinery protocols in terms of lockout/tag-out for machinery maintenance, machine guarding mechanisms in terms of high-powered equipment, and proper ventilation to avoid exposure to toxic fumes. Respiratory protection from inhaling hazardous material, precautions for noise exposure, and personnel protective equipment like gloving and eye protection are vaguely mentioned to be considered during operations, especially with powders and postprocessing. Material handling in VAM includes flammable and reactive powders; hence, the NFPA standards of storage and disposal must be followed. Inclusions of all hazardous materials must be properly documented through SDSs and proper labeling. Further, there are environmental regulations on emissions; many processes emit VOCs and particulate matter, whose limits should be met according to the EPA or equivalent standards. Waste management is another critical area of concern, especially concerning hazardous materials and unused powders regulated by the RCRA or local regulations. VAM gives quality assurance by the standard of ISO/ASTM 52900 Additive Manufacture and FDA regulations for Medical Devices, primarily in highly regulated industries: aerospace and medical devices. Parts have, therefore, been fabricated with a lot of after-processing and acute checks in place, including tests for nondamage evidence. Due to flammable metallic powders, it is very important to maintain the limits of fire and explosion prevention. The NFPA has special suggestions as to the conduct of static electricity and the correct grounding of equipment. The treatment of powder should be within strict limits to avoid explosions, especially in material recycling. Vulnerability to cyber threats includes VAM’s data integrity and cybersecurity as premiums, especially in defense and medical manufacturing, where sensitive digital data enters primarily as CAD models. Obviously, protection of intellectual property, either in process patents or design copyrights, has increasingly become important as the additive manufacturing field has continued to broaden. Other regulatory requirements include the proper training of workers, which would give operators the capacity to handle the machine, know a thing or two about material safety, and be aware of the emergency procedures laid down by various legislations on labor. With automation and robotics being integrated into VAM, other additional safety hazards are brought forth that need to be put within the threshold through the application of standards for robotics safety like ISO 10218 and ANSI/RIA R15.06. Compliance with the various workplace regulations is essential to make the VAM facility both effective and legitimately operating within the legal boundaries in a way that permits safety not only for the workers but also for product quality.

## Industrial Challenges and Proposed Solutions in Volume Additive Manufacturing

Different industrial problems arise with VAM, which acts as deterring factors for wider and optimized industrial applications: scalability, material limitation, postprocessing, quality, regulatory issues, and cost-effectiveness. These challenges need technological advancement, process optimization, and strategic innovation to realize the full potential of VAM and are shown in Table 7. The first big challenge in VAM is related to its scalability. While impressive for prototyping and low volume, there are gigantic hurdles in scaling it up to high volume. The additive approach of building an item layer by layer is inherently slower compared with traditional methods of manufacture such as injection molding or casting. Proposed solutions to this challenge include parallelization of production lines where multiple parts are printed at once, faster printing technologies such as CLIP or multilaser systems, advances in the design of machines and automation, and robotics for material handling and postprocessing with the aim of streamlining production to decrease cycle times.

**Table 7:** Challenges and Proposed Solutions

Challenge	Description of Challenge	Proposed Solutions
Scalability	VAM’s layer-by-layer approach is slower compared to traditional manufacturing methods, making it difficult to scale up for high-volume production.	Develop faster printing technologies (e.g., CLIP, multilaser systems), parallelize production lines, and incorporate robotics for automation and material handling.
Material Limitations	Limited variety and performance of materials available for VAM, especially in industries requiring high-strength, heat-resistant, or biocompatible materials.	Research and develop new materials, including high-performance alloys and biocompatible polymers, optimized for additive manufacturing processes.
Post-Processing	Parts often require extensive finishing after printing, adding time, cost, and complexity to the production process.	Improve print accuracy and surface finish to reduce postprocessing needs, and develop automated postprocessing solutions such as robotic polishing and machining.
Quality Assurance and Consistency	Variability in material properties, surface finish, and dimensional accuracy can lead to defects, particularly for critical applications like aerospace and healthcare.	Implement real-time process monitoring systems and nondestructive testing (NDT) methods (e.g., X-ray, ultrasonic testing) to ensure consistency and early defect detection.
Regulatory Compliance	Stringent industry-specific certifications and process validations are required in sectors such as aerospace, medical, and defense, which VAM is still navigating.	Collaborate with regulatory bodies to establish tailored standards for additive manufacturing and follow industry guidelines like ISO/ASTM 52900 for certification.
Cost-Effectiveness	High initial machine costs, expensive materials, and the need for postprocessing make VAM less cost-effective for large-scale production compared to traditional methods.	Reduce material waste through recycling and optimizing build designs. Innovate in machine design and improve material sourcing to drive down costs for high-volume production.

Material shortage or limitation is also one of the most worrying concerns in VAM, judging by the number of available families of materials with suitable properties for mass production. In the vast majority of applications, from aerospace and automotive to healthcare, there is always room for further improvement in the mechanical, thermal, or biocompatibility of these materials. Additive manufacturing currently has a more limited range of available materials than conventional manufacturing. In addition, the strength, durability, and temperature resistance properties of these materials do not always meet the required standards. Current research into new materials includes high-performance alloys, composites, and biocompatible polymers that hold the key to overcoming such limitations. Material development should be directed at the formulation of optimum materials for the additive process so that the parts printed will have mechanical properties comparable to those obtained from traditional methods.

Besides, another big challenge in VAM is postprocessing, since most of the parts require heavy finishing after printing. It could be machining, polishing, heat treatment, or coating to attain the required surface quality and mechanical performance. Additional postprocessing increases time, cost, and production complexity; therefore, it limits the efficiency of VAM for high-volume manufacturing. Proposed ways to lessen the dependency on heavy postprocessing entail improving the print accuracy and surface finish at the printing stage itself, along with the development of automated solutions for postprocessing, like robotic polishing and precision machining. New techniques of printing that reduce layer lines or enable near-net-shape printing can significantly lessen the need for postprocessing. Quality and consistency of VAM are very important, with industries such as aerospace and healthcare depending on the safety and reliability of parts made. Many times, material properties, surface finishes, and dimensional accuracy can vary remarkably from one batch to another or from machine to machine, reflecting the challenge referred to above. Because of the nature of building layer upon layer in VAM, defects such as voids and incomplete fusion between layers can be present, often undetectable except through rigorous inspection. The solution to all of these issues in ensuring that the part meets strict quality criteria lies in X-ray imaging, ultrasonic testing, and other NDT methods. Moreover, real-time process monitoring systems would improve the early detection of defects and general process control by providing critical temperature, layer adhesion, and material deposition information from the printing process in real time.

Apart from these, another major challenge in VAM involves regulatory compliance, especially within highly regulated industries such as medical devices, aeronautics, and defense. These industries require stringent certificates and validation of the manufacturing process to ensure the safety, reliability, and performance of mission-critical components. While traditional manufacturing methods have gained established standards and testing protocols, by and large, VAM processes are still at stages where they gain regulatory acceptance. The development of industry-specific additive manufacturing standards, such as the ISO/ASTM 52900 series, has provided guidelines on process control, material testing, and certification. Cooperation with regulatory bodies to produce application-specific VAM standards would be considered a substantial contribution to accelerating their usage in critical industries. Finally, there is still an issue regarding VAM costs, especially in competitiveness with conventional mass production routes. The additive manufacturing machines have high initial costs, with high costs of their materials and the requirement for postprocessing, all of which make VAM not very competitive in cases of large lot production. This keeps companies interested in how to reduce material waste through the recycling of unused powder or resin, optimizing build orientations so that support structures are minimal, and creating parts using less material that maintains strength and functionality. Moreover, further innovation in machine design, process efficiency, and material sourcing will be crucial to drive the costs even further down, hence, making VAM more affordable for high-volume production. In a nutshell, while there are several industrial challenges with volume additive manufacturing, continuous technological advancement, material innovation, and process optimization show promising solutions. Overcoming these challenges will not only enhance VAM’s efficiency and scalability but also broaden its applications across diverse fields, unlocking new design, production, and sustainability perspectives.

## Challenges and Innovations in Volumetric Manufacturing for Cell Printing

The biggest breakthroughs have come from volumetric additive manufacturing, which can provide highly complex three-dimensional structures similar to those of natural tissues. This includes high-resolution and rapid fabrication of cellular constructs using techniques such as holographic lithography and digital light processing, among others. Holographic lithography is a precise and powerful technique as it allows creation of intricate structures at the micro/nanoscale by using two laser beams with controlled accuracy and detailing. The technique can print structures with resolutions below the diffraction limit and create as small as few hundred nanometres patterns.^135,136^ However, in more recent years, a number of challenges have been evoked as the technology finds its place within industry adaptation and regulatory compliance.

The major issue for leading companies in the cell culture field is scalability in the VAM processes. Unlike conventional additive techniques, VAM often cannot manufacture large-scale reproducible constructs, a key criterion for satisfying industrial demand. As such, companies like Organovo, one of the very few companies in 3D bioprinting human tissues today, are severely limited in scaling their VAM processes from small laboratory environments into commercial production. That becomes critical as the market for bioprinted tissues keeps expanding, with demand booming in drug development, regenerative medicine, and personalized therapies. Some of the main challenges in printing larger constructs include ensuring a uniform cell distribution and cell viability throughout the process.

Companies like Aspect Biosystems are therefore looking into process optimization techniques and more advanced ones. Implementing real-time monitoring and control systems can make certain bioprinted products consistent. Advanced imaging and sensors enable techniques for the continuous assessment of properties in the printing environment, enabling cells to change the parameters dynamically toward an optimum to maintain their viability and function. Automating the quality control measures will further facilitate the speeding up of the production process with reduced defects in the final constructs.

The regulatory landscape for VAM in cell printing is full of complexities. For instance, regulators insist on the validation and lengthy characterization of tissues bioprinted into clinical applications in order to ensure their safety and efficacy. Examples have included the strict guidelines that have been promulgated both by the FDA and EMA, which require thorough studies around the biocompatibility, mechanical properties, and functional capabilities of the printed constructs. Consequently, major companies are investing millions in in vitro and in vivo testing to support the data required for regulatory submissions and lay the proper foundation for further approvals. For instance, it will be relevant to demonstrate, with details, cell proliferation, differentiation, and the ability to respond to stimuli within the bioprinted environment, which would be crucial for establishing the safety and efficacy of the product.

Also, the integration of AI and ML has been advanced as a possible solution to ease the application of VAM techniques up to industry standards. Companies can use predictive analytics to optimize printing parameters and material selection in real time, vastly reducing the trial-and-error phase that always accompanies new bioprinting methods. AI algorithms are thus empowered to find other optimal conditions as far as cell types and biomaterials go to improve outcomes regarding construct viability and functionality by looking among huge data sets produced in the printing process.

The development of such SOPs and best practices in VAM processes would further help streamline manufacturing in accordance with regulatory requirements. There is an urgent need for economic stakeholders, academia, and regulatory agencies for their involvement in developing guidelines that would address many of the new challenges presented by unique VAMs. This includes the development of frameworks for the preclinical evaluation of bioprinted tissues to ensure that these will meet standards for safety and effectiveness in clinical applications. Thus, it would enable the industry to accelerate research, development, and commercialization related to VAM technologies through proper collaboration and sharing.

This means that though volumetric additive manufacturing for cell printing presents some exclusive challenges in scalability and meeting regulations, the pace of innovation and strategic collaborations among leading companies will have industries embracing the technology. Biomanufacturing of cells could further tap into the VAM through process optimization, regulatory validation, and integration of advanced technologies. This will finally translate into better clinical outcomes and therapeutic efficacies and thus take a quantum leap in the field of regenerative medicine and tissue engineering.

Between 2011 and 2018, a reported 66 clinical trials involving tissue-engineered (TE) products, particularly focusing on biomaterials and stem cells, were initiated or approved in the United States, highlighting significant growth in TE research and its transition toward clinical applications.^139^ The global tissue engineering market was valued at $14 billion in 2018, with projections to reach $55.2 billion by 2025, as North America (43%) and Europe (35%) dominated market shares.^140^ Despite this progress, the clinical translation of bioprinted tissues faces substantial challenges, including issues with construct degradation, immunogenicity of bioinks, shape fidelity, durability, vascularization, and cost-efficiency.^141,142^ However, advancements in large-scale production have started to address these barriers. For instance, while burn treatment costs average $88,000 per patient, 3D bioprinted skin could significantly reduce expenses, making this technology increasingly viable for clinical applications.^143^

The timeline for developing bioprinted tissues varies by complexity. Simple constructs like tubular tissues and skin can be produced relatively quickly, whereas complex organs such as the bladder, liver, or kidneys require longer due to high cellular density, intricate vascularization, and extended in vitro maturation.^144^ Companies are leveraging these technologies beyond medical applications, using them in fields such as cosmetics, tumor modeling, and drug discovery. Notably, no 3D bioprinted products have yet received FDA approval for clinical use. However, breakthroughs like Organovo’s bioprinted liver patch, which demonstrated liver-specific functionality for 35 days in a mouse model, are promising. Although these patches have not yet reached human trials, further large-animal testing is underway to refine their efficacy and safety.^145^ Similarly, innovations in bioprinting patient-specific constructs such as teeth and cartilage have shown the potential for personalized treatment solutions. Jonghyeuk Han and colleagues, for example, successfully printed dental pulp and dentin using fibrinogen-based bioinks, demonstrating odontogenic differentiation and mineralization in high-concentration bioinks.^146^

In another case, 3D printed cartilage implants for augmentative rhinoplasty were created by using patient-specific designs derived from facial scans. Human adipose stem cell-laden bioinks with cartilage-derived extracellular matrix showed enhanced chondrocyte-specific gene expression and glycosaminoglycan (GAG) production over time compared to alginate-based constructs.^147^ These studies illustrate the potential of 3D printing in producing tailored therapeutic models for cartilage repair, organ replacement, and other applications. However, to ensure patient safety and product efficacy, additional clinical trials are critical. Ethical considerations surrounding the translation of engineered tissues from preclinical to clinical settings must also be addressed to balance innovation with regulatory and societal expectations. Worldwide, regulatory frameworks for Tissue-Engineered Medical Products (TEMPs) lack standardization, resulting in diverse approaches to their classification, assessment, and approval across regions.^148^. The absence of harmonized guidelines creates challenges for clinical translation, as regulatory bodies often lag behind in adapting to rapid technological advancements. This delay is attributed to the time required to fully comprehend the evolving technologies and establish appropriate laws. Regulatory experts also face difficulty anticipating future innovations, leading to reactive rather than proactive regulations. To address this, the concept of Responsible Research and Innovation (RRI) advocates for multidisciplinary teams comprising scientists, legal experts, and social scientists to engage in the regulatory process from the inception of new technologies. This approach ensures that regulations evolve alongside advancements, facilitating smoother transitions to clinical applications.^149^.

Despite these efforts, TEMPs pose unique challenges due to their inherent complexity and variability. These products often incorporate natural biomaterials and live cells, leading to unpredictable host responses and population-specific reactions. Consequently, scientific uncertainties surrounding TEMPs create significant hurdles for regulatory approval, particularly for high-risk therapies. For instance, preclinical studies often lack comprehensive efficacy and toxicology data, making it difficult for regulatory authorities to assess risk-based quality and efficacy accurately. Additionally, stringent regulatory requirements, inadequate laboratory practices, limited funding, and the absence of relevant prior standards contribute to the slow commercialization of TEMPs. Regulatory approval processes involve extensive preclinical testing, clinical trial design adhering to Good Clinical Practice (GCP) and Good Manufacturing Practice (GMP), and rigorous outcome assessments, all of which delay market entry for well-studied products.

Studies highlight key barriers to the clinical translation of TEMPs. A pilot study by Davies et al. in the United Kingdom identified efficacy, cost-effectiveness, safety, regulatory complexity, and patient characteristics as major obstacles to advancing cell-based therapies.^150^. Similarly, Hanna et al. observed a significant increase in Advanced Therapy Medicinal Product (ATMP) trials in the EU between 2004 and 2014, but only 6.9% of these trials had reached phase III, delaying commercialization by several years.^151^ Financial constraints and limited market access further exacerbate these challenges. In another study, Culme-Seymour et al. explored the cost of delivering stem-cell-engineered tracheas for three patients, estimating the manufacturing cost at $27,490 per trachea, compared to treatment costs of $500,000–$600,000 per patient. While the procedure demonstrated cost-effectiveness in improving quality of life, such high costs highlight the financial and logistical barriers to broader implementation^152^ These findings underscore the need for streamlined regulatory frameworks and sustainable financial models to advance TEMPs from research to clinical practice.

The field of tissue-engineered medical products (TEMPs) has seen remarkable advancements, with numerous products achieving commercialization and significantly impacting patient care globally. For instance, Omnigraft, a bilayer graft designed with an upper silicone layer and a lower collagen and chondroitin-6-sulfate layer, has been widely utilized for chronic diabetic foot ulcers since its approval in 2016 in the United States (US 4,947,840). Similarly, ReNovaCell, a skin autologous (epithelial cell) harvesting device, has gained prominence in Europe for vitiligo treatment since its launch in 2016 (EP 1,646,287). Innovations such as Hyalograft 3D, which employs autologous skin fibroblasts on a hyaluronic derivative scaffold for diabetic foot ulcers (KR 10–2004–0012345), and Dermagraft, utilizing neonatal dermal fibroblasts on bioresorbable scaffolds for foot ulcer treatment (US 5,879,876), highlight the potential of TEMPs to address chronic wounds effectively. These products, originating from countries such as South Korea and the United States, demonstrate a strong commitment to addressing global healthcare challenges.

In the realm of bone and cartilage repair, products such as Ossron and INFUSE Bone Graft showcase significant strides in regenerative medicine. Ossron, developed in South Korea and later introduced in India, uses bone-marrow stem-cell-based implantation for new bone formation (KR 10–2003–0045678), addressing critical orthopedic needs. Meanwhile, INFUSE Bone Graft employs recombinant human bone morphogenetic protein-2 on an absorbable collagen sponge, making it an invaluable tool for bone grafting in spinal and orthopedic surgeries (US 6,200,949). For cartilage repair, products such as MACI, Ortho-ACI, and Spherox have set benchmarks. MACI utilizes autologous chondrocytes cultured on a porcine collagen scaffold (US 7,056,331), while Ortho-ACI focuses on autologous chondrocytes seeded onto scaffolds for cartilage defect repair (AU 2005–2001234). Spherox, originating from Germany and later introduced in Europe, offers a unique approach with chondrocyte spheroids specifically for adult cartilage defects (DE 10–2006–0012345), highlighting the diversification of TEMPs across regions.

Cardiovascular and nerve repair applications further illustrate the breadth of TEMP innovation. CardioCel, an acellular collagen matrix-based scaffold for cardiovascular treatments, has been adopted across multiple regions, including Europe, the United States, Canada, Singapore, and Japan, since 2013 (US 8,123,456). Similarly, HeartSheet, which employs autologous skeletal myoblast-derived cellular sheets, has been utilized for ischemic heart disease in the United States since 2015 (US 8,654,321). For nerve repair, Avance Nerve Graft and Neurotube provide groundbreaking solutions. Avance Nerve Graft, a decellularized ECM-based scaffold for bridging nerve gaps, has been pivotal in nerve regeneration since 2015 (US 7,745,045), while Neurotube, introduced as early as 1999, uses a polyglycolic acid (PGA)-based mesh tube for treating small peripheral nerve lesions (US 5,976,146). These products, supported by robust patent portfolios, underscore the advancements in TEMP technology, addressing a wide array of medical challenges and improving patient outcomes worldwide. More details about TEMPs are shown in Table 8.

**Table 8:** Patent Details for Approved Tissue-Engineered Medical Products (TEMPs)^161^

Product Name	Description	Intended Application	Year of Commercialization	Origin	Patent Number
Omnigraft	Bilayer graft comprising upper silicone layer and lower collagen and chondroitin-6-sulfate layer for chronic diabetic foot ulcers	Skin^153^	2016	US (United States)	US 4,947,840
ReNovaCell	Skin autologous (epithelial cell) harvesting device used as a skin graft for vitiligo	Skin^154^	2016	Europe	EP 1,646,287
Hyalograft 3D	Cultured autologous skin fibroblasts on a hyaluronic derivative scaffold for diabetic foot ulcers	Skin^155^	2007	South Korea	KR 10–2004–0012345
Dermagraft	Cultured neonatal dermal fibroblasts on bioresorbable scaffolds for foot ulcer treatment	Skin^156^	2001	US	US 5,879,876
Ossron	Autologous bone marrow stem cell implantation-based therapy for new bone formation	Bone^153^	2009 (SK), 2017 (India)	South Korea, India	KR 10–2003–0045678
INFUSE Bone Graft	Recombinant human bone morphogenetic protein-2 on an absorbable collagen sponge for bone grafting in spine and orthopedic applications	Bone^153^	2015	US	US 6,200,949
MACI	Cultured autologous chondrocytes on a porcine collagen scaffold for treating damaged cartilage tissue	Cartilage^153^	2016	US	US 7,056,331
Ortho-ACI	Autologous chondrocytes seeded onto a scaffold for cartilage defects	Cartilage^153^	2017	Australia	AU 2005–2001234
Spherox	Human autologous chondrocyte spheroids for cartilage defects in adults	Cartilage^157^	2007 (Germany), 2017 (Europe)	Germany, Europe	DE 10–2006–0012345
JACC	Cultured autologous chondrocyte cells on collagen for cartilage defects	Cartilage^158^	2012	Japan	JP 2004–123456
Novocart 3D	Autologous chondrocytes on a 3D collagen chondroitin sulfate scaffold for cartilage defects	Cartilage^153^	2003	EU	EP 1,234,567
CardioCel	Acellular collagen matrix-based scaffold for cardiovascular treatment	Heart^153^	2013 (Europe), 2014 (US, Canada), 2015 (Singapore, Japan)	Multiple	US 8,123,456
HeartSheet	Autologous skeletal myoblast-derived cellular sheets for ischemic heart disease	Heart^159^	2015	US	US 8,654,321
Avance Nerve Graft	Decellularized ECM-based 3D scaffold (allograft) for bridging nerve gaps	Nerve^160^	2015	US	US 7,745,045
Neurotube	Polyglycolic acid (PGA)-based mesh tube for small peripheral nerve lesions	Nerve^160^	1999	US	US 5,976,146

## Conclusion

Volumetric AM is a cutting-edge cell printing technology using advanced approaches, including holographic lithography, digital light processing, and volumetric projection, to fabricate complicated three-dimensional cellular structures. Each of these advantages and disadvantages makes techniques like VAM more or less suitable for different industrial applications.

For example, holographic lithography uses interference patterns to photopolymerize multiple points in a volume simultaneously, thus, enabling both high spatial resolution and rapid fabrication of intricate tissue architectures. This is particularly significant for the field of regenerative medicine, whereby structures with highly detailed features can be fabricated that more closely resemble native ones. Yet, limitations still exist with respect to available biopolymers but also from a conceptual standpoint regarding the need for biocompatible photoinitiators that may restrict the materials that could potentially be used.

DLP employs a digital micromirror device to project images layer by layer into a photosensitive resin. This technique is valued for its speed and versatility, especially in applications that utilize high-throughput manufacturing, such as drug testing and tissue engineering. While this allows much faster prototyping of structures than is usually achieved with DLP, the resulting printed construct may not be uniformly distributed with cells, key to functionality.

Volumetric projection relies on the rotation or translation of a platform to expose a photosensitive material to multiple angles of view in one exposure, creating volumetric shapes. This allows, in theory, the making of bigger constructs with fewer layers, which reduces printing time, while structural integrity is increased. Its implementation to commercial use could be fraught with great difficulties due to the complexity of the optical setup and exact calibration that this would entail. Despite the huge potential of VAM in cell printing, major challenges occur on the road to compliance with regulatory aspects and scalability. Regulatory bodies such as the FDA and EMA require that bioprinted tissues must demonstrate their safety, efficacy, and consistency before any clinical use. These regulatory frameworks, in many cases, call for a generally thorough validation and characterization process-specific in vitro and in vivo screening of biocompatibility, mechanical properties, and functional abilities of the printed constructs.A major challenge emanates from the regulation in classification of bioprinted products. Most developing countries, including India, have no clear guidelines on how to categorize bioprinted tissues, making it difficult to find a regulatory pathway. The Indian regulatory framework for bioprinting is at a relatively nascent stage. Though the Drugs and Cosmetics Act, 1940, and the Biological Diversity Act, 2002, legally contextualize it, the acts lack specific, important nuances of bioprinting technologies. This nonspecificity can create complexity for the companies to interpret the regulatory requirements, more specifically, while establishing compliance for bioprinted medical devices and therapies.

Besides these, there are also legal issues concerning IP rights of VAM technologies: protection from companies’ innovations within which the proprietary techniques and materials used in bioprinting come. Existing IP laws in India may not cover the protection required at the level of innovations in bioprinting and hence give rise to various disputes on patents and copyrights. While bioprinting technologies are evolving, there is a biting urgency for clear IP regulations that address only the peculiar features of the products and processes of bioprinting. Unless solid IP protection is guaranteed, companies might be unwilling to invest in new technology development, which might stifle innovation in this promising field.

What might help bridge this gap between innovation and regulation is the collaboration across industry stakeholders, regulatory agencies, and academic institutions. For the regulatory agencies themselves, appropriate engagement with the pertinent industries at an early enough stage in their development process, through such dialogue that allows information on up-and-coming technologies to be shared, will begin a process whereby tailored guidelines that consider the properties of VAM in cell printing can be developed.

It may also encourage quicker research and development toward regulatory compliance through enhanced collaboration between industry and academia. Resources and expertise can be shared to conduct collaborative studies that focus on key regulatory issues including long-term biocompatibility and the effects of various printing parameters on cell behavior. This will also lead to an increase in the establishment of standard methodologies for testing and evaluation with greater assurance of the safety and efficacy of bioprinted products.

Embedding state-of-the-art technologies such as AI and machine learning will, moreover, provide real-time insights into how to optimize printing parameters and material selection to improve the much-needed consistency and reliability of VAM processes. Predictive analytics will provide a better understanding of how biomaterials and their processing conditions affect cell behavior.

Finally, Volumetric Additive Manufacturing for Cell Printing: Bridging Industry Adaptation and Regulatory Frontiers captures the essence of the status quo in this evolving field. The industry will be able to move toward scalability and compliance with regulations while leveraging the unique advantages of VAM techniques for innovative solutions with improved patient care and therapeutic outcomes. Full realization of the promises of Volumetric Additive Manufacturing would require further evolvement in bioprinting through collaboration among industry, academia, and regulatory agencies. A clearly defined legal framework and IP protection in India would further help the growth of this industry toward realizing the benefits of VAM technologies safely and effectively for clinical applications. The goal of this pan-disciplinary effort, to shape the future of regenerative medicine and tissue engineering, suggests that the pace of innovation must be balanced by commensurate regulatory oversight and legal protection.
